# Enhanced anti-tumor effect of liposomal Fasudil on hepatocellular carcinoma *in vitro* and *in vivo*

**DOI:** 10.1371/journal.pone.0223232

**Published:** 2019-10-03

**Authors:** Ying Zhao, Yu Zhang, Milad Vazirinejad Mehdiabad, Ke Zhou, Yuyuan Chen, Lei Li, Jun Guo, Chuanrui Xu

**Affiliations:** 1 Department of Pharmacy, Union Hospital, Tongji Medical College, Huazhong University of Science and Technology, Wuhan, China; 2 School of Pharmacy, Tongji Medical College, Huazhong University of Science and Technology, Wuhan, Hubei, China; Chung Shan Medical University, TAIWAN

## Abstract

Hepatocellular carcinoma (HCC) is one of the most malignant cancers and the treatment options for this disease are limited and generally not effective. ROCK has been reported to be highly expressed in many cancer types and its inhibitor Fasudil has shown anti-cancer potential. However, its high toxicity and low solubility restrict its clinical application. Here, we report that Fasudil is effective against HCC and that a liposomal formulation (Lip-Fasudil) can enhance the anti-tumor effects of this drug both *in vitro* and *in vivo*. *In vitro*, Fasudil inhibited HCC cell growth with IC_50_ values of 0.025–0.04 μg/μL, with Lip-Fasudil showing slightly improved cytotoxicity with IC_50_ values of 0.02–0.025 μg/μL. Cellular mechanistic analysis indicated that Fasudil induced cell cycle arrest at the G2/M phase and that Lip-Fasudil enhanced this effect. Intriguingly, no apoptosis was detected in Fasudil- or Lip-Fasudil-treated HCC cells. *In vivo*, Fasudil inhibited the growth of HCC xenografts by 23% in nude mice. However, Lip-fasudil exerted anti-tumor effects (57% tumor inhibition) that were superior to those of Fasudil and similar to those of Topotecan (66%). In addition, Lip-fasudil resulted in an increased distribution of Fasudil in tumor tissues but a reduced distribution in normal organs. In conclusion, our results proved that Fasudil has the potential to be used for HCC treatment and that a liposomal formulation (Lip-Fasudil) could enhance anti-tumor efficacy and reduce systemic toxicity.

## Introduction

Hepatocellular carcinoma (HCC) is the most common primary liver tumor and one of the most important lethal malignancies worldwide [1, 2]. In China, the incidence of HCC has been increasing continually [3] and the 5-year survival rate has been less than 15% in the past decade [4, 5]. Currently, optional treatments for liver cancer are very limited and the prognosis is generally unsatisfactory [6–8]. Sorafenib is the only targeted drug approved for the treatment of unresectable liver cancer at present. However, it can only improve the survival of HCC patients by 2–3 months [9, 10]. Therefore, exploring novel drugs is an urgent need for HCC treatment.

ROCK kinase inhibitors have been widely studied for many types of diseases, and in the 1990s, one such agent, Fasudil, was shown to increase both cerebral blood flow and glucose metabolism. Subsequently, it was approved for the prevention of post-operative cerebral vasospasm and the treatment of subarachnoid hemorrhage in Japan [11–13]. In addition, Fasudil improves p53-mediated apoptosis in human HCC cells [14]. Currently, it is mainly used clinically for the treatment of hypertension, coronary vasospasm, atherosclerosis, pulmonary hypertension, aortic sclerosis, heart failure-related vascular resistance, contraction, cerebral arterial spasm, ischemic stroke, and kidney transplant [13, 15]. However, in 2006, Han *et al*. proved that Fasudil could inhibit tumor progression using three independent animal models [16]. This result provided a good foundation for the study of Fasudil as an anticancer drug. Further, in 2009, Yang *et al*. confirmed that Fasudil could inhibit the migration of 95-D lung cancer cells [17]. In addition, they proved that it could suppress tumor growth by inhibiting Rho/Rho kinase signaling, resulting in the downregulation of ABCE1, which regulates the migration of 95-D cells. In 2013, Peng *et al*. also confirmed that Fasudil could inhibit proliferation after abdominal aortic aneurysm and maintain tumors at their initial size [18]. The mechanism underlying the anti-tumor effect was attributed to damage to the tumor cell membrane. This damage might lead to the infiltration of macrophages along with the angiogenesis. Together, these results suggest that Fasudil has potential for the treatment of different cancers.

However, the anti-tumor effect of Fasudil on HCC had not been fully evaluated. In addition, since Fasudil can act on normal tissues including vasculature and the heart [19–21], its distribution in tumor tissues needs to be improved to reduce side effects and improve its efficacy. In this study, we prepared a liposomal preparation of this drug (Lip-Fasudil) to enhance its distribution in tumor tissues through the EPR effect (enhanced permeability and retention). Then, we examined its cytotoxicity and anti-tumor mechanism in HCC cells, as well as its anti-tumor effect in HCC mouse models.

## Materials and methods

### Materials

Fasudil, cholesterol (Chol), L-glutamate, and Sepharose CL-4B chromatography media were purchased from Sigma-Aldrich Chemical Co (St. Louis, MO, USA). DMEM high glucose medium was bought from Hyclone of Thermo Scientific (IL, USA). Trypsin was purchased from Beyotime Institute of Biotechnology (Beijing, China). 1, 2-dioleoyl- 3-trimethylammonium-Propane (Chloride Salt) (DOTAP) and monomethoxy polyethylene glycol 2000-distearoyl phosphatidyl-ethanolamine (mPEG-DSPE) were purchased from Avanti Polar Lipid (Alabaster, AL, USA). PD-10 desalting columns were purchased from GE Healthcare Biosciences (PA, USA). All reagents and solvents were of analytical or HPLC grade and were used without further purification.

### Preparation of Fasudil liposomes

The cationic liposomes were prepared by the thin film hydration and polycarbonate membrane extrusion method according to the literature [22, 23]. DOTAP/Chol/mPEG-DSPE was used at a molar ratio of 40:55:5, respectively. Fasudil was remote loaded into the liposomes by a transmembrane pH gradient. Briefly, the total lipids (85 mg) were dissolved in CHCl_3_ and dried to a thin film by rotary evaporation followed by further drying under a vacuum at 40 °C. The lipid film was then hydrated with 2 mL (NH_4_)_2_SO_4_ (250 mM) for 30 min at 60 °C [24]. The resultant multilamellar vesicles were extruded five times through 0.2-μm pore-size polycarbonate membranes and five times through 0.1-μm pore-size polycarbonate membranes using a Lipex Extruder (Northern Lipids Inc., Canada) driven by pressurized nitrogen at 60 °C to produce homogeneous unilamellar vesicles. The residual (NH_4_)_2_SO_4_ was removed with phosphate-buffered saline (PBS, pH 7.4) by size-exclusion chromatography on a PD-10 column. Fasudil (Sigma Chemical Co., St Louis, MO, USA) was loaded into the liposomes at a phospholipid:Fasudil ratio of 1:0.4 (w/w) and incubated for 1 h at 65 °C. Free Fasudil was separated from Lip-Fasudil by size exclusion chromatography on a Sepharose CL-4B column equilibrated with 25 mM HEPES and 140 mM NaCl (HEPES buffer, pH 7.4) [25, 26].

### Size and zeta potential

The particle size and zeta potential of the liposomes were measured by Zeta PALS (Zeta Potential Analyzer, Brookhaven Instruments Corporation, Austin, TX) according to the manufacturer’s instructions. All measurements were carried out at room temperature. Each parameter was measured three times, and average values and standard deviations were calculated.

### Cell culture

HepG2, Huh7, Hep3B, and SMMC-7721 cells were all from the China Center for Type Culture Collection at Wuhan University (Wuhan, China). These cells were cultured with high glucose DMEM medium supplemented with penicillin, streptomycin and 10% FBS in 37 °C and 5% CO_2_ incubators. M-plasmodia (San Diego, CA, USA) at a concentration of 2.5 μg/mL was used to prevent possible mycoplasma infections.

### Mice

Female BALB/c nude mice (6–8 weeks of age, 16–18 g) and female FVB/N mice (6–8 weeks of age, 18–20 g) were obtained from Huafukang Technology Corporation (Beijing, China) and kept in filter-topped cages with standard rodent chow and water available ad libitum and a 12-h light/dark cycle. The experiments were performed according to national regulations and approved by the Animal Experiments Ethical Committee of Huazhong University of Science and Technology.

### Cytotoxicity

The cytotoxic effect of Lip-Fasudil and free Fasudil against the HCC cells was measured using a Cell Counting Kit-8 kit (CCK-8 kit, Dojindo laboratories, Kumamoto, Japan). HepG2, Huh7, Hep3B, and SMMC-7721 cells were separately seeded into 96-well plates at a density of 5 × 10^3^ cells/well. After 24 h, cells were treated with Lip-Fasudil and free Fasudil. Cells were further incubated for 24 h and the proportion of viable cells was measured calorimetrically according to the user’s manual. Cell viability within each group was expressed as a percentage of the viability of control cells.

### Cell apoptosis analysis

For cell apoptosis analysis by FACS, HCC cells were seeded in DMEM with 10% FBS in 6-well plates (1.5 × 10^5^ cells per well) and allowed to attach overnight. The medium was then changed to fresh DMEM with 10% FBS and cells were treated with PBS (control), free Fasudil (0.02 μg/μL), or Lip-Fasudil (0.02 or 0.03 μg/μL). At the end of treatment for 24 h, cells were harvested in PBS and stained with the Annexin V-FITC Apoptosis Detection kit I (BD Pharmingen TM, San Diego, CA) according to the manufacturer’s instructions. They were then analyzed within 1 h by flow cytometry using a FACS array apparatus (BD Biosciences) with Cell Quest (BD Biosciences) and Mod Fit LT (Verify software, Topsham, MN) software.

### Cell cycle analysis

For cell cycle analysis, HCC cells were seeded in DMEM with 10% FBS in 6-well plates (1.5 × 10^5^ cells per well) and allowed to attach overnight. The medium was then changed to fresh DMEM with 10% FBS and cells were treated with PBS (control), free Fasudil (0.02 μg/μL), or Lip-Fasudil (0.02 or 0.03 μg/μL) for 24 h. Next, cells were digested with trypsin and collected with the suspension. After fixation with 70% ethanol, cells were incubated with 0.5 mg/mL RNase and 0.025 mg/mL propidium iodide for 30 min. For each sample, 5 × 10^4^ cells were detected by flow cytometry (CytomicsTM FC 500, Beckman Coulter, USA) and the results were analyzed using software FlowJo 7.6 (FlowJo, USA).

### Western blot analysis

For western blot analysis, HCC cells were seeded in DMEM with 10% FBS in 6-well plates (1.5 ×10^5^ cells per well) and allowed to attach overnight. The medium was then changed to fresh DMEM with 10% FBS and cells were treated with PBS (control), free Fasudil (0.02 μg/μL), or Lip-Fasudil (0.02 or 0.03 μg/μL) for 24 h. Next, cells were lysed in Cell Lysis Reagent (Sigma-Aldrich, MA, USA) for 30 min on ice, and the supernatant was collected after centrifugation at 12,000 g. Cell lysates were separated on a 10% acrylamide gel and transferred to a PVDF membrane. Membranes were blocked for 1 h in 5% skim milk and then incubated with primary antibody overnight. Membranes were washed in TBST (PBS with 0.1% tween-20) three times and then incubated for 1 h with secondary antibody. Then, the membranes were washed four times and developed by an enhanced chemiluminescence system according to the manufacturer’s instructions (Perkin Elmer, Waltham, MA). The antibodies used for Western blotting is listed in Table 1.

**Table 1:** Primary antibodies used for western blotting.

Name	Mono/Poly	Host Species	Source	Catalogue Number	Dilution
Cyclin A2	Poly	Rabbit	Proteintech	18202-1-AP	1:1000
CyclinB1	Poly	Rabbit	Proteintech	55004-1-AP	1:1000
CyclinE1	Poly	Rabbit	Proteintech	11554-1-AP	1:1000
CyclinD1	Mono	Mouse	Proteintech	60186-1-Ig	1:1000
Bcl2	Mono	Rabbit	CST	3498	1:1000
Bax	Poly	Rabbit	CST	2772	1:1000
β-actin	Mono	Rabbit	CST	4970	1:1000
AKT	Mono	Rabbit	CST	4691	1:1000
p-AKT(s473)	Mono	Rabbit	CST	4060	1:1000
ERK(1/2)	Mono	Rabbit	CST	4695	1:1000
p-ERK(1/2) (Thr202/Tyr204)	Mono	Rabbit	CST	4370	1:1000
GAPDH	Poly	Rabbit	Proteintech	10494-1-AP	1:1000

### Xenograft mouse model

For subcutaneous implantation, 4 × 10^6^ Hep3B cells were injected per nude mouse. After implantation, tumor growth was observed every other day and tumor volumes were calculated as V = 0.52 × a × b^2^, with “a” representing the long diameter and “b” the short diameter. At a tumor volume of ~100 mm^3^, liposome or free drugs were injected as a multiple intravenous bolus via intraperitoneal injection at a dose of (30 mg/kg separately) every other day for a total of 10 times. The tumor sizes were measured every 3 days and any animal death was recorded.

### Tissue distribution

Mouse stomach, hearts, livers, spleens, kidneys and tumor tissues were collected at 0.25, 1, 3, and 6 h after injection. Tissues of 0.1 g were homogenized with 500 μL saline and then centrifuged (Eppendorf5415D, 12,000 g, 15 min) at 4 °C to collect the supernatant. Then 200 μL of supernatant was added to 200 μL methyl alcohol and 400 μL DMSO and the mixture was vortexed for 30 s and centrifuged at 4 °C (12000 × *g*, 10 min) to separate the Fasudil-containing supernatant from the tissue protein. The absorption of Fasudil standard solutions and samples in tissues were measured by HPLC at an excitation wavelength of 276 nm (High performance liquid chromatograph Waters, American). The concentration of Fasudil in each sample was calculated by using as standard curve determined by a series of Fasudil dilutions.

### Hydrodynamic injection, free Fasudil, and Lip-Fasudil treatment

For hydrodynamic injections, 25 μg of the plasmids carrying the genes (*Akt* and *RAS* in pT3-EF1a) with a sleeping beauty transposase (pCMV-SB) at a ratio of 25 μg: 25 μg: 5 μg were diluted in 2 mL saline (0.9% NaCl) for each mouse. Saline solution was filtered through a 0.22-μm filter and then injected into the lateral tail vein of 6–8-week-old FVB mice over 5–7 s. After 1 week post-injection, liposomal or free drugs were injected via i.p. at a dose of 50 mg/kg for 3 weeks. All plasmids were gifts from Dr. Xin Chen (University of California at San Francisco). All animal experiments were approved by the Animal Care and Use Committee at Huazhong University of Science and Technology.

### Immunohistochemistry

Livers were fixed in 4% paraformaldehyde and embedded in paraffin. For immunohistochemistry, deparaffinized sections were incubated in 3% H_2_O_2_ dissolved in 1× phosphate-buffered saline (PBS) for 30 min to quench the endogenous peroxidase. For antigen retrieval, slides were microwaved in 10 mM citrate buffer (pH 6.0) for 10 min. Subsequently, slides were incubated with primary antibodies overnight at 4 °C. All primary antibodies used in the present investigation were selected among those that were previously validated by the manufacturers for immunohistochemistry. The immunoreactivity was visualized with the Vectastain Elite ABC kit (Vector Laboratories, Burlingame, CA). The specificity of primary antibody reactivity was confirmed by either omitting the primary antibody in the immunohistochemical procedure or, when available, by incubating the primary antibody for 2 h at room temperature with its specific blocking peptide in a 1:2 dilution, before adding the primary antibody to the slides. Slides were counterstained with Mayer’s hematoxylin. The antibodies used for IHC is listed in Table 2.

**Table 2:** Primary antibodies used for immunohistochemistry.

Name	Mono/Poly	Host Species	Source	Catalogue Number	Dilution
p-AKT(s473)	Mono	Rabbit	CST	4060	1:200
HA	Poly	Rabbit	Proteintech	51064-2-AP	1:100
N-Ras	Poly	Rabbit	Proteintech	10724-1-AP	1:200
Ki67	Poly	Rabbit	Abcam	Ab15580	1:200

### Humane endpoints

All 12 nude mice and nine FVB mice were monitored every day during the study. Monitoring included weight, activity, sizes of tumor (nude mice), or degree of abdominal swelling (FVB mice). All control mice and most treated mice were euthanized by cervical dislocation immediately when the control group mice had a body condition score of 2 or less and a low activity with large tumors (nude mice) or a noticeable swelling abdominal mass (FVB mice). In addition, the remaining treated mice were used for survival analysis and were sacrificed at specific days after control mice were euthanized. The weight, liver weight, tumor size, and date of sacrifice were recorded.

### Statistical analysis

All *in vitro* experiments were repeated three times and in vivo experiments were repeated two times. All statistical analyses were performed using Graph Pad Prism 5.0 software. The differences between groups were compared using a Student’s t-test and data were expressed as the mean ± SD. Values of P < 0.05 were considered significant.

## Results

### Preparation and characterization of Lip-Fasudil

**Fig 1:**
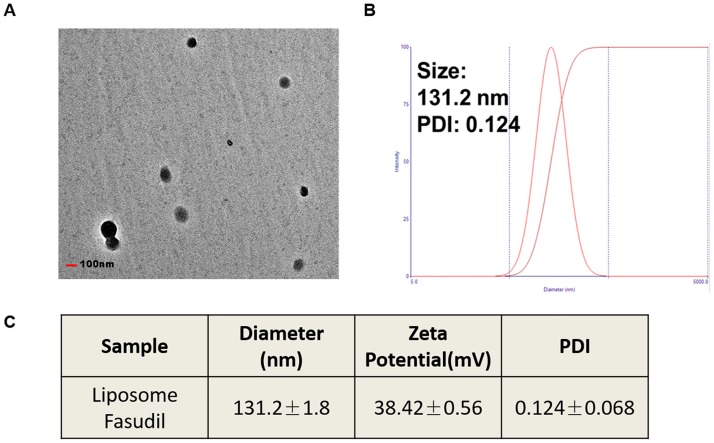
Preparation and characterization of Lip-Fasudil. (A) Morphology of Lip-Fasudil, as detected by TEM; (B) size distribution of Lip-Fasudil; (C) zeta potential of Lip-Fasudil. Experiments were repeated three times and data are expressed as the mean ± SD (n = 3). Scale bar = 100 nm.

Lip-Fasudil was prepared according to the methods mentioned in the materials and methods. Examination by transmission electron microscopy showed that the liposomes were round and dispersed well in water solution (Fig 1A). As determined by dynamic light scattering, the average particle size of Lip-Fasudil was 131.2 nm (Fig 1B) and the zeta potential was 38.42 mV (Fig 1C). The polydispersity index was 0.124, suggesting an even distribution of particle sizes (Fig 1C). These results indicated the Lip-Fasudil was prepared with uniform shapes and sizes.

### Both Fasudil and Lip-Fasudil exert cytotoxic effects against HCC cells

**Fig 2:**
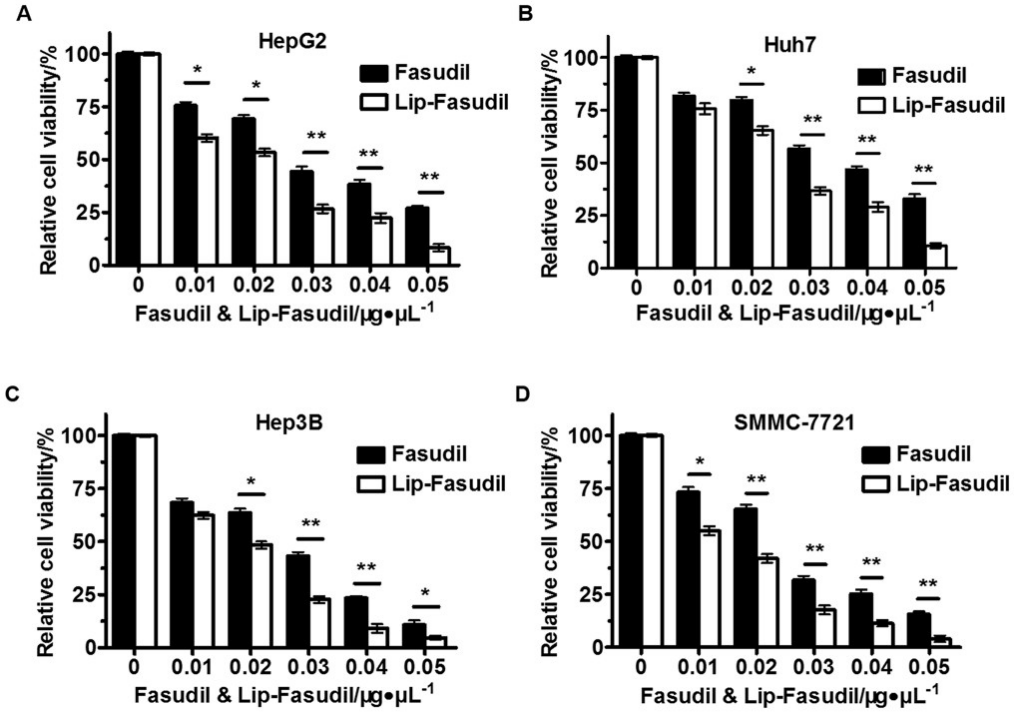
Cytotoxic effects of Lip-Fasudil and free Fasudil against four hepatocellular carcinoma (HCC) cell lines. Cells were treated with free Fasudil and Lip-Fasudil at concentrations of 0, 0.01, 0.02, 0.03, 0.04, and 0.05 μg/μL for 24 h. A no treatment group was used as a negative control. Data from the groups were then compared by one-way ANOVA with a Dunnett’s post-test. Data are expressed as the mean ± SD (n = 3). *p < 0.05 and **p < 0.01.

To examine the cytotoxicity of Lip-Fasudil and free Fasudil using four HCC cell lines, we examined their activities at different concentrations (0, 0.01, 0.02, 0.03, 0.04, and 0.05 μg/μL) for 24 h. In HepG2, Huh7, Hep3B, and SMMC-7721 cells, IC_50_ values of free Fasudil were 0.03, 0.04, 0.03, and 0.025 μg/μL, respectively, whereas those of Lip-Fasudil were approximately 0.02, 0.025, 0.02, and 0.02 μg/μL, respectively (Fig 2). Treatment with free Fasudil for 24 h significantly inhibited the growth of all four HCC cell lines. Likewise, Lip-Fasudil showed comparable cytotoxicity with free Fasudil after 24 h of treatment (Fig 2). Therefore, both Fasudil and Lip-Fasudil showed anti-tumor activity against HCC cells and that of Lip-Fasudil was higher than that of free Fasudil. In addition, increasing the dose of Fasudil or Lip-Fasudil was found to augment cell death, suggesting that the cytotoxicity of Fasudil and Lip-Fasudil was dose-dependent in these cell lines.

### Fasudil and Lip-Fasudil kill HCC cells independent of apoptosis

**Fig 3:**
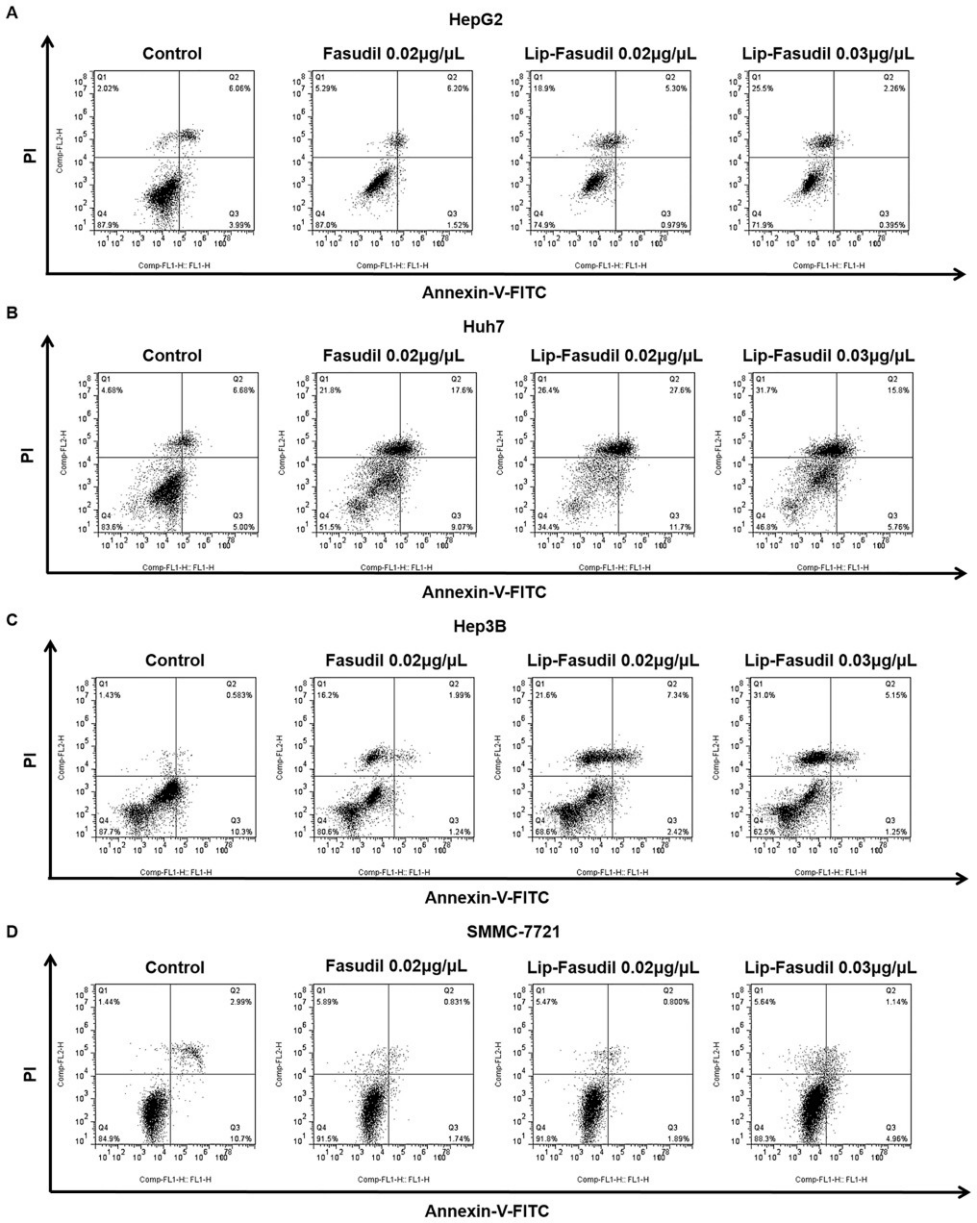
Free Fasudil and Lip-Fasudil do not induce apoptosis. HepG2 (A), Huh7 (B), Hep3B (C), and SMMC-7721 (D) cells were treated with 0.02 μg/μL free Fasudil, 0.02 μg/μL Lip-Fasudil, or 0.03 μg/μL Lip-Fasudil for 24 h and then collected for flow cytometric analysis. Quadrants from lower left to upper left (counter clockwise) represent healthy, early apoptotic, late apoptotic, and dead cells, respectively. The percentages of cells in each quadrant are shown on the graph.

To investigate the anti-tumor mechanism underlying the effects of Fasudil and Lip-Fasudil on HCC cells, we evaluated their impact on cell apoptosis and cell cycle progression. Cells were collected after 24 h of drug treatment and flow cytometry (FACS) was performed. In HepG2, Huh7, Hep3B, and SMMC-7721 cells, free Fasudil induced apoptosis in only 6.20%, 17.60%, 1.99%, and 0.83% cells, respectively. In comparison, 0.02 μg/μL Lip-Fasudil resulted in apoptosis in 5.30%, 27.60%, 7.34%, and 0.80% cells, respectively (Fig 3). These results showed that in HCC cells, the induction of cellular apoptosis was not a major mechanism through which both free Fasudil and Lip-Fasudil inhibited HCC growth.

However, we observed visible morphological changes in cells. FACS results showed that both Fasudil and Lip-Fasudil increased non-apoptotic cell death in HepG2 cells. Free Fasudil induced non-apoptotic death in approximately 5.3% of cells, whereas 0.02 and 0.03 μg/μL Lip-Fasudil resulted in non-apoptotic death in 18.9% and 25.5% of cells (Fig 3A). These results suggested that Lip-Fasudil increases non-apoptotic cell death in a dose-dependent manner. Similar results were observed with Huh7, Hep3B, and SMMC-7721 cells. Free Fasudil induced non-apoptotic cell death in approximately 21.80%, 16.20%, and 5.89% of Huh7, Hep3B, and SMMC-7721 cells, respectively. In comparison, 0.02 μg/μL Lip-Fasudil resulted in rates of 26.4%, 21.60%, and 5.47%, and 0.03 μg/μL Lip-Fasudil induced non-apoptotic death in 31.70%, 31.00%, and 5.64% of cells, respectively (Fig 3B, 3C and 3D). In those cells, both free Fasudil and Lip-Fasudil did not increase the proportion of apoptotic cells significantly, but profoundly increased the incidence of non-apoptotic cell death. Taken together, these results demonstrated that Fasudil exerts anti-tumor activity by increasing non-apoptotic cell death and that Lip-Fasudil shows enhanced anti-tumor effects, as compared to Fasudil, by promoting this activity.

### Fasudil and Lip-Fasudil induce non-apoptotic programmed cell death by blocking cell cycle progression

**Fig 4:**
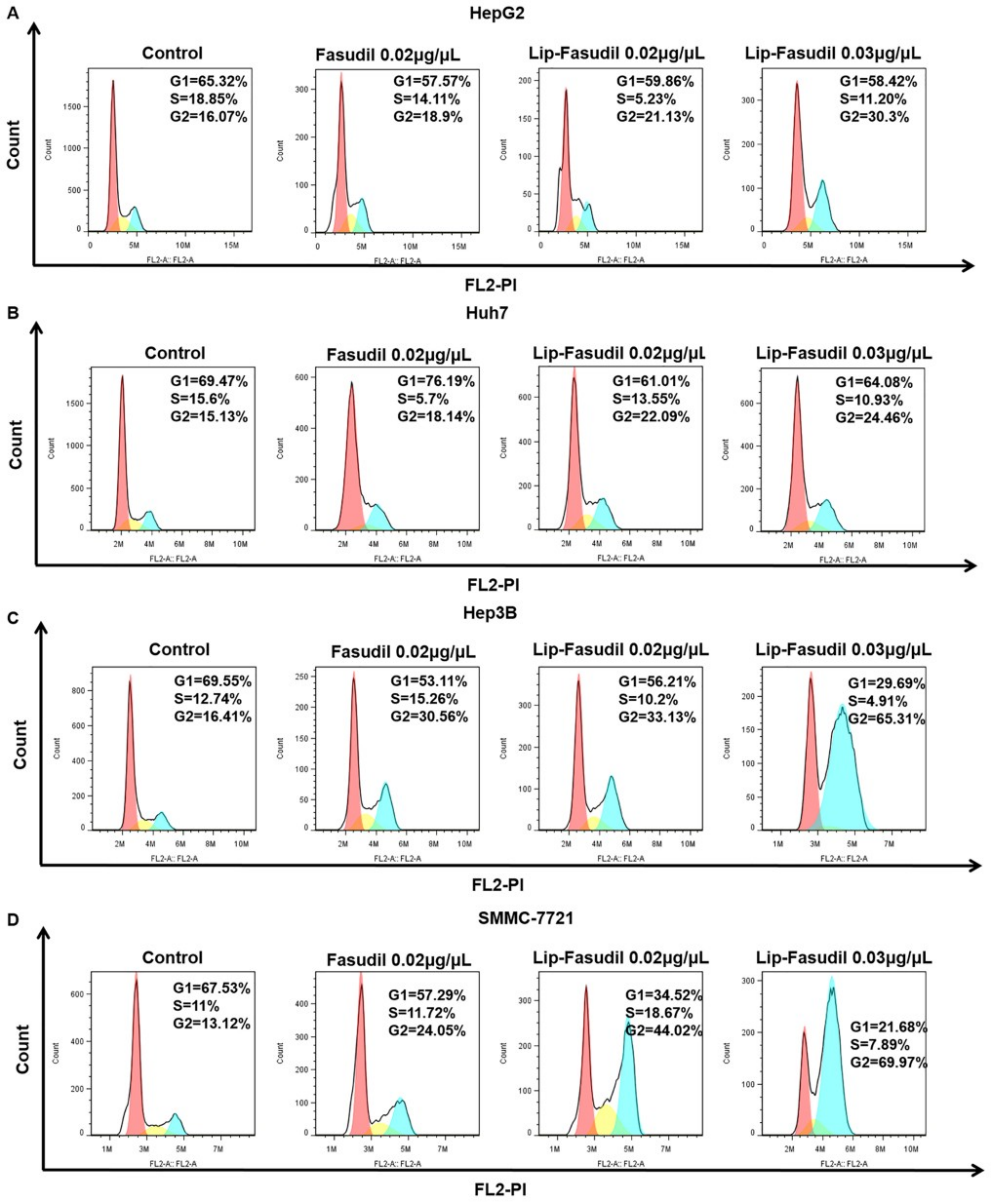
Fasudil and Lip-Fasudil induce cell cycle arrest. HepG2 (A), Huh7 (B), Hep3B (C), and SMMC-7721 (D) cells were administrated Fasudil or Lip-Fasudil at indicated doses (Fasudil, 0.02 μg/μL; Lip-Fasudil, 0.02 and 0.03 μg/μL) for 24 h. Then, cells were analyzed by flow cytometry for cell cycle distribution; 2 × 10^4^ cells were detected in each test.

To investigate how Fasudil or Lip-Fasudil induced non-apoptotic programmed cell death in HCC cells, we performed flow cytometric analysis to examine cell cycle progression after drug treatment. Flow cytometry results showed that free Fasudil resulted in an increased ratio of G2/M phase HepG2 cells (18.90%) and that Lip-Fasudil treatment led to the accumulation of cells in G2/M phase (21.13%) compared to that with free Fasudil. More importantly, with increasing concentrations of Lip-Fasudil, the ratio of HepG2 cells in G2/M phase (30.30%) was also increased (Fig 4A). The same effect was observed in Huh7, Hep3B, and SMMC-7721 cells. In these cells, proportions of cells in the G2/M phase were 15.13%, 16.41%, and 13.12%, respectively. Further, after treatment with 0.02 μg/μL free Fasudil, the proportions of cells in the G2/M phase were increased to 18.14%, 30.56%, and 24.05%, respectively. In contrast, 0.02 μg/μL Lip-Fasudil treatment increased cells in the G2/M phase to 22.09%, 33.13%, and 44.02%, respectively. Further, 0.03 μg/μL Lip-Fasudil treatment increased these proportions to 24.46%, 65.31%, and 69.97%, respectively (Fig 4B, 4C and 4D). These results suggested that Fasudil blocks cell cycle progression at G2/M phase in HCC cells and then induces non-apoptotic cell death.

**Fig 5:**
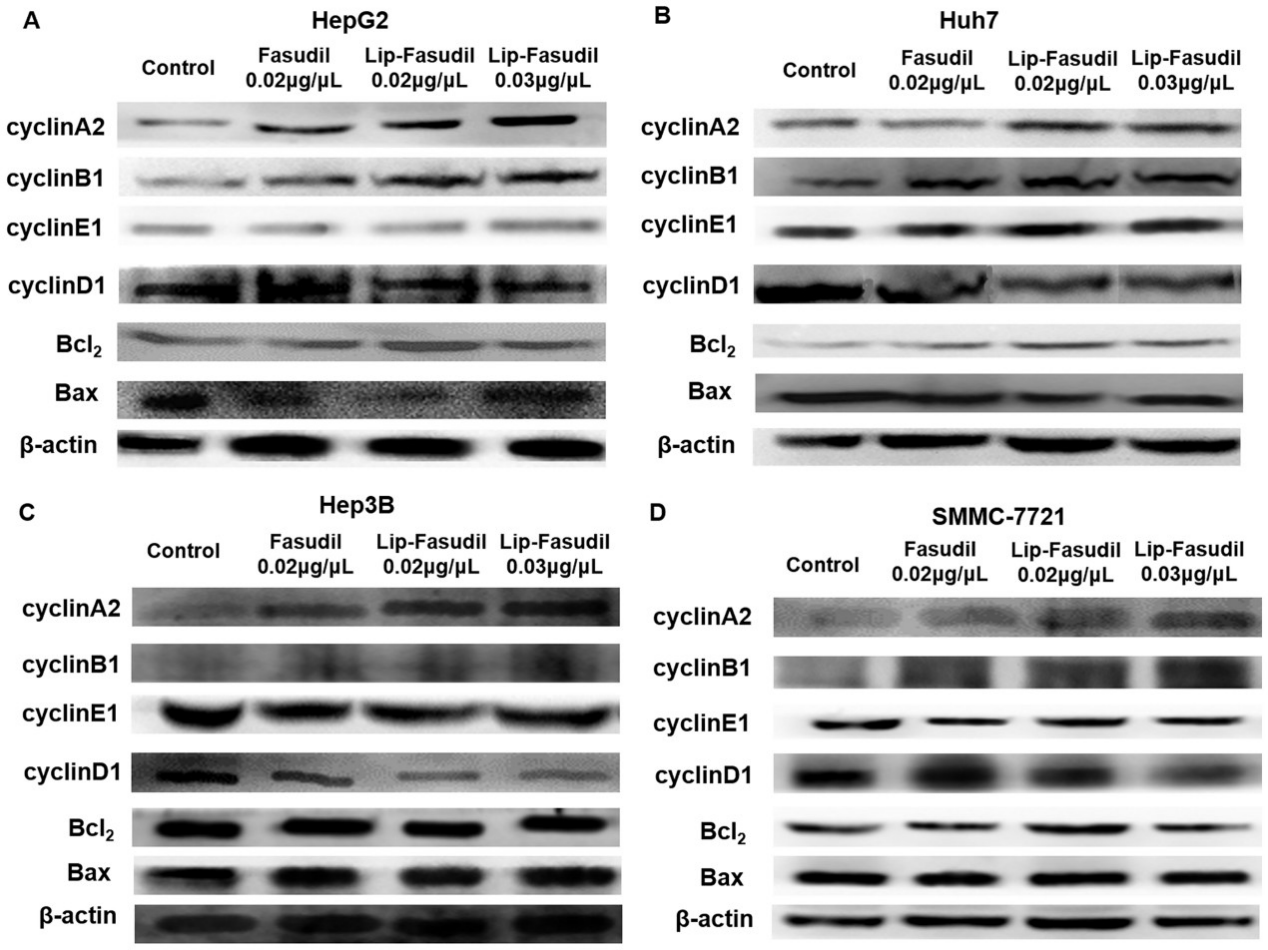
Detection of cell cycle- and apoptosis-related proteins by western blotting after Fasudil treatment. Hepatocellular carcinoma (HCC) cells were treated with 0.02 μg/μL Fasudil or 0.02 or 0.03 μg/μL Lip-Fasudil for 24 h and then harvested for western blotting analysis. Protein expression levels of ROCK2, cyclin A2, cyclin E1, cyclin B1, cyclin D1, Bcl_2_, and Bax were detected in HepG2 (A), Huh7 (B), Hep3B (C), and SMMC-7721 (D) cells. β-actin was used as a loading control.

To further confirm this conclusion, we detected the expression of cell cycle- and apoptosis-related proteins in drug-treated cells by western blotting. The result showed that Fasudil and Lip-Fasudil could markedly reduce ROCK2 expression (Fig 5). Moreover, in HepG2, Huh7, Hep3B, and SMMC-7721 cells, expression levels of the apoptosis-related protein BCL2 did not change significantly after the administration of Fasudil and Lip-Fasudil (Fig 5); however, the expression of Bax was marginally increased in HepG2 cells (Fig 5A). In addition, cyclin D1 was downregulated upon Fasudil and Lip-Fasudil treatment. Cyclin A2 and cyclin B1 also exhibited higher expression levels due to cell cycle blockade in the G2/M phase (Fig 5). These results were consistent with the flow cytometric results indicating that Fasudil could induce cell cycle arrest at the G2/M phase. In addition, cell cycle arrest caused by Lip-Fasudil was more remarkable than that with Fasudil.

### Lip-Fasudil shows enhanced anti-tumor effects against tumor xenografts

**Fig 6:**
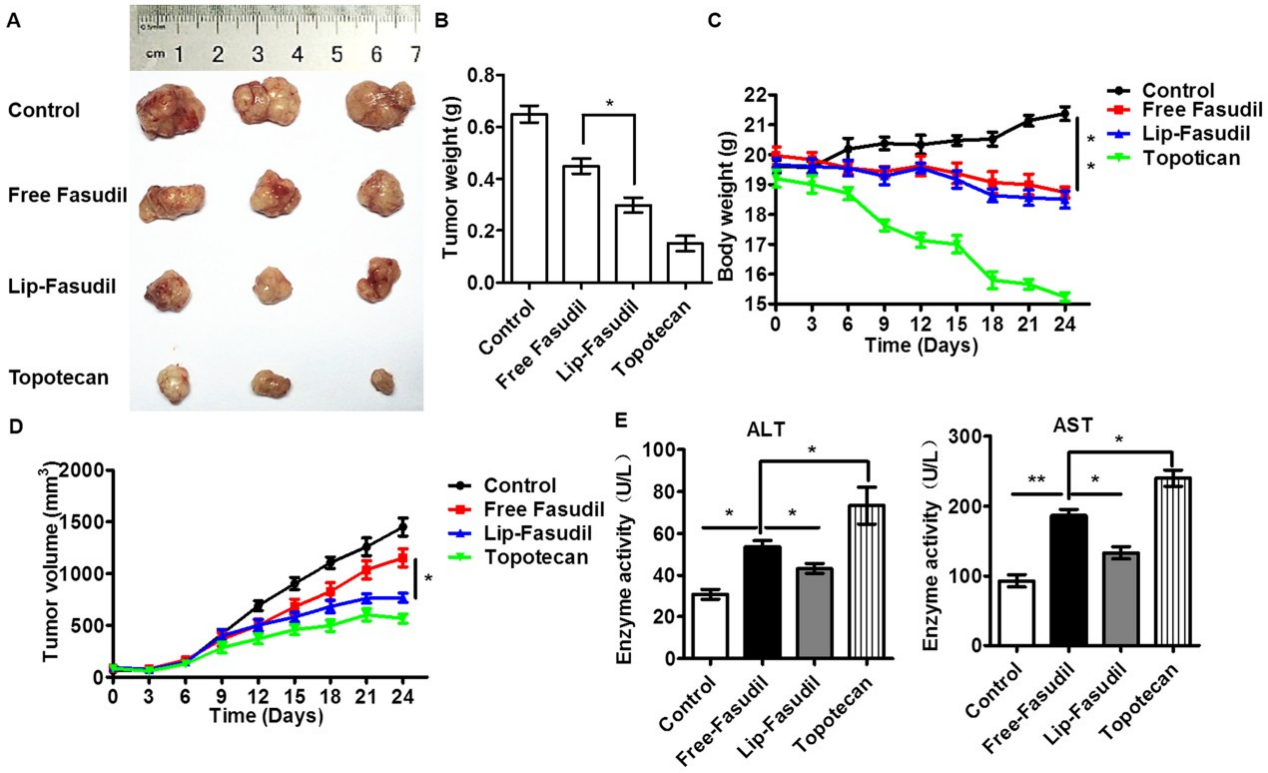
Anti-tumor effects of free Fasudil and Lip-Fasudil in a xenograft mouse model. (A) Excised tumors at the end point of the experiment. (B) Weight of tumors at the end point of the experiment. (C) Growth curves of xenograft tumors after treatment with saline, free Fasudil (30 mg/kg), Lip-Fasudil (30 mg/kg), and Topotecan (2 mg/kg) by a single dose via tail vein injection. Data are expressed as the mean ± SD (n = 3). (D) Body weight changes in mice treated with saline, free Fasudil, Lip-Fasudil, and Topotecan. Saline injection was used as the control. (E) Aminotransferase (AST) and alanine aminotransferase (ALT) levels in mice at the end of the experiment. The sera (n = 3) were collected after mice were sacrificed, and ALT and AST levels were determined using an automatic biochemical analyzer. Data are expressed as the mean ± SD (n = 3). *p < 0.05, **p < 0.01 compared with free Fasudil group.

The anti-tumor efficacies of Fasudil and Lip-Fasudil were further evaluated using a Hep3B xenograft tumor model. Fasudil and Lip-Fasudil were administrated by intraperitoneal injection when the subcutaneous tumor grew to ~100 mm^3^. The injection was conducted every other day for a total of 10 doses. On the 24th day after the first injection, the mice were sacrificed, and tumors were collected for volume determinations. Compared to that in the saline group (0.62 g, 1450 mm^3^), free Fasudil treatment (0.43g, 1120 mm^3^) inhibited tumor growth by 23% (Fig 6A and 6B). Lip-Fasudil (0.36g, 626 mm^3^, 57%) showed an enhanced anti-tumor effect compared to that with free Fasudil, but its effect was decreased compared to that of Topotecan (0.16g, 488 mm^3^, 66%) (Fig 6A and 6B). Body weight examination also showed that the tumor burden in Lip-Fasudil-treated mice was much less than that in free Fasudil-treated mice (Fig 6C). Tumor growth curves showed that the anti-tumor effects of free Fasudil and Lip-Fasudil were due to continuous growth inhibition induced by these formulations (Fig 6D). Of note, Topotecan resulted in significantly increased levels of aspartate aminotransferase (AST) and alanine aminotransferase (ALT) levels, indicating liver injury in mice (Fig 6E). In comparison, free-Fasudil-treated mice had lower AST and ALT levels than those treated with Topotecan, indicating diminished hepatic toxicity. Interestingly, Lip-Fasudil resulted in a further reduction in hepatic toxicity. Collectively, these data suggested that Fasudil exerts anti-tumor effects in a xenograft tumor model and that Lip-Fasudil has enhanced anti-tumor effects and reduced systemic toxicity.

### Lip-Fasudil increases the accumulation of Fasudil in tumor tissue

**Fig 7:**
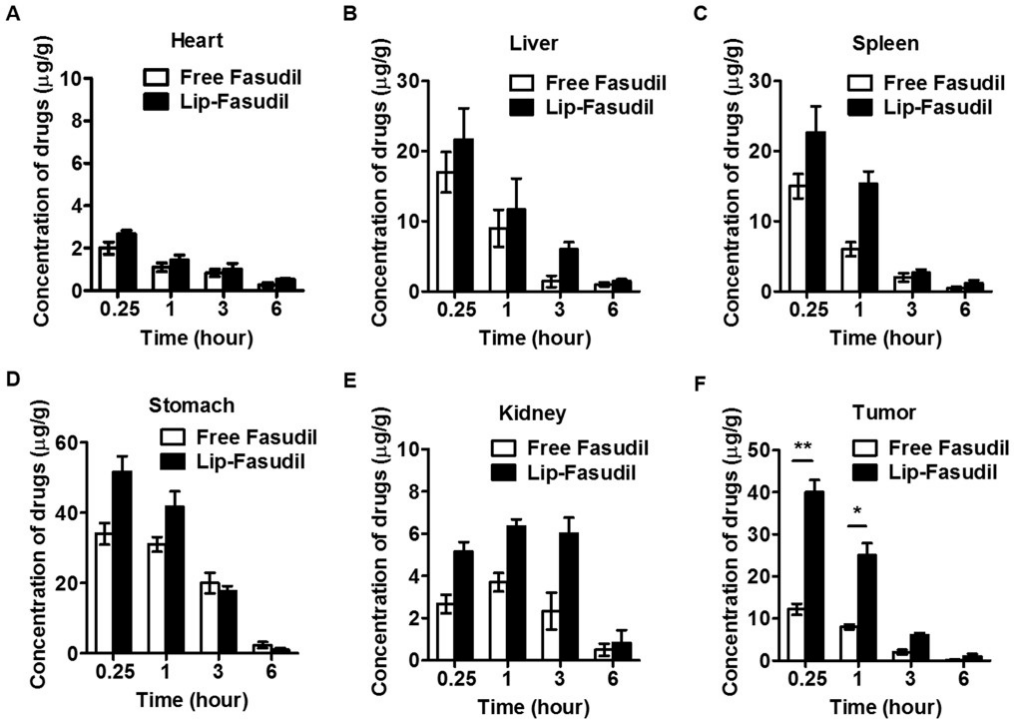
Distribution of free Fasudil and Lip-Fasudil in xenograft mouse model. Mice were injected (i.p) with free Fasudil (30 mg/kg) or liposomal Fasudil (30 mg/kg), and tissues were collected at 0.25, 1, 3, and 6 h post-injection. Organs (A to E) or tumor (F) samples were homogenized and drugs were extracted to analyze the Fasudil content using HPLC. Data are presented as the average ± standard error (n = 3), and the statistical significance level was *p < 0.05, **p < 0.01.

According to the aforementioned results, we inferred that the liposomal preparation (Lip-Fasudil) could increase the distribution of Fasudil in tumor tissues. To compare the distribution of free Fasudil and Lip-Fasudil in mouse tissues, we injected tumor-bearing mice with each preparation and then examined their distribution. Compared to that with free Fasudil, Lip-Fasudil significantly increased the amount of Fasudil in the mouse liver, spleen, stomach, and kidney at 0.25 h after injection (Fig 7A–7E). Further, the amount of free Fasudil in tumor tissue was significantly lower than that of Lip-Fasudil at 0.25, 1, and 3 h after injection (Fig 7F). These results indicated that Lip-Fasudil could increase the accumulation of Fasudil in tumor tissues through EPR effects.

### Lip-Fasudil improves the anti-tumor effect of free Fasudil in AKT/Ras-induced HCC model

**Fig 8:**
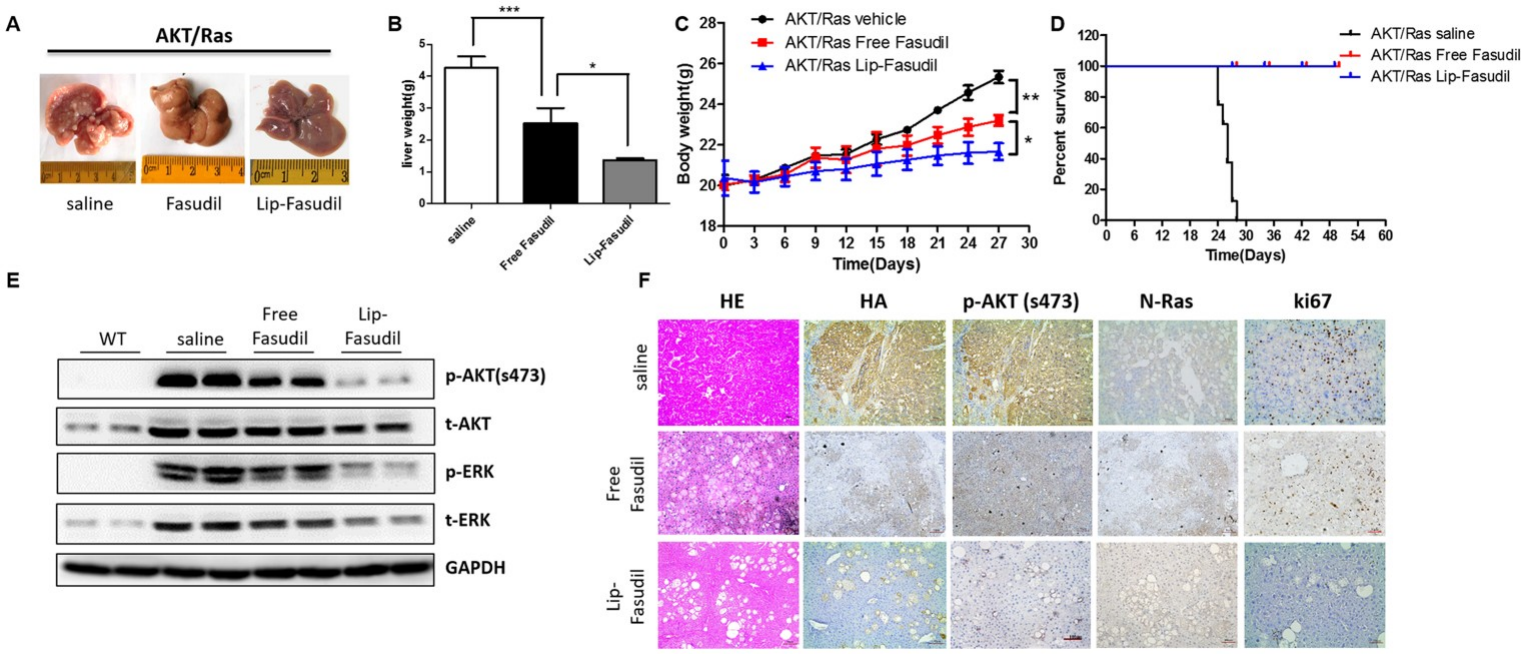
Anti-tumor effects of free Fasudil or Lip-Fasudil in hepatocellular carcinoma (HCC) mouse model induced by AKT and N-Ras oncogenes. Liver gross image (A), liver weight (B), body weight (C) and survival curve (D) of mice that treated with saline, free Fasudil (50 mg/kg) or Lip-Fasudil (50 mg/kg). (E) Protein expression of p-AKT (S473), t-AKT, p-ERK and t-ERK in WT, AKT/Ras saline, AKT/Ras free Fasudil and AKT/Ras Lip-Fasudil liver tissues, GAPDH was used as loading control. (F) Immunohistochemistry analysis of H&E, HA, p-AKT, N-Ras and Ki67 in different groups. Data are presented as average ± standard error (n = 3), and the statistical significance level is *p < 0.05, **p < 0.01 and ***p < 0.001.

We then explored the therapeutic potential of Fasudil and Lip-Fasudil in primary mouse HCC models. An Akt/Ras-induced primary HCC mouse model was established by the hydrodynamic injection of plasmids carrying the Akt and Ras oncogenes into mice, as previously described [27]. One week after Akt/Ras injection, free Fasudil and Lip-Fasudil were injected (i.p) at a dose of 50 mg/kg every day for 3 weeks. At 4.2 weeks after hydrodynamic injection, all mice were sacrificed. Many nodules were observed on the liver in the control group. However, fewer nodules were observed in Fasudil and Lip-Fasudil group mice. Interestingly, the enhanced anti-tumor effect of Lip-Fasudil was confirmed by observed decreases in liver and body weight (Fig 8A, 8B and 8C). Survival curves also indicated that Lip-Fasudil and free Fasudil could prolong the life of HCC mice by more than 4 weeks (Fig 8D). In addition, expression levels of p-AKT (S473), AKT, p-ERK, and ERK in different groups were determined by western blotting and immunohistochemical staining. The results showed that both AKT (with a HA tag) and Ras were expressed in AKT/Ras-injected mouse liver tissues (Fig 8E and 8F). However, Fasudil and Lip-Fasudil reduced the levels of AKT, p-AKT, Erk, and p-Erk, downstream effectors of Ras signaling, concomitant with reduced tumor growth. Moreover, the decrease in Ki67 staining in treated liver tissues demonstrated that Fasudil could suppress tumor cell proliferation and that Lip-Fasudil could further enhance this effect (Fig 8F). Taken together, these results showed that Fasudil is effective against HCC and that Lip-Fasudil can enhance its anti-tumor effects by improving its distribution in tumor tissues.

### Discussion

HCC is a serious threat to human health, and the incidence of this disease has been increasing for decades. However, drugs approved for HCC treatment are extremely limited. In this study, we explored the anti-tumor activity of Fasudil in HCC cells and a mouse model. Our results clearly demonstrated that it has anti-tumor activity for HCC *in vivo* and *in vitro*. Thus, our study provides evidence for the potential application of Fasudil in HCC treatment.

We further demonstrated the anti-tumor effect of Fasudil in HCC cells. *In vitro*, both Fasudil and Lip-Fasudil could inhibit the growth of HCC cells by blocking cell cycle progression and promoting non-apoptotic programmed cell death. However, neither free Fasudil nor Lip-Fasudil induced apoptosis in four HCC cell lines. However, both induced cell cycle arrest in HCC cells and then induced non-apoptotic programmed cell death. The effect of Fasudil on cell cycle progression was possibly due to its inhibition of Rho kinase. The inhibition of Rho kinase blocks actomyosin contractility by dephosphorylation and the activation of myosin light chain phosphatase [28]. Several studies have revealed that the Rho/ROCK pathway plays a crucial role in modulating cell skeleton reorganization via myosin light chain phosphorylation [29]. Hence, Fasudil might block cell cycle progression by inhibiting the formation of spindle bodies during cellular mitosis.

In addition, our study investigated the feasibility of using liposomes to deliver Fasudil and demonstrated that Lip-Fasudil had significantly enhanced anti-tumor effects, compared to those with Fasudil, with reduced systemic cytotoxicity. Since Doxil, the first liposomal pharmaceutical product, was approved by the FDA in 1995, liposomes have been intensively investigated for applications in clinics. Many types of liposomes including PEGylated liposomes (Doxil, Lipo-dox), temperature-sensitive liposomes (ThermoDox), cationic liposomes (EndoTAG-1), and virosomes (Epaxal and Inflexal V) have been tested clinically or developed for clinical use (at different stages) [30]. Accumulating evidence has demonstrated that liposomal formulations can prolong blood circulation time, vary drug distribution in the body, and thus reduce possible side effects related to the drugs (e.g., cardiotoxicity). However, liposome formulations are not suitable for all drugs and they have some limitations. For example, in comparison with Doxil, ThermoDox resulted in significantly diminished doxorubicin accumulation in mouse tumors 24 h after administration [31]. In this study, we found that liposomes can significantly increase the uptake and accumulation of Fasudil in tumor tissues. Correspondingly, Lip-Fasudil significantly enhanced the anti-tumor effect of Fasudil in xenograft and primary HCC models, even though it showed only slightly higher anti-tumor activity than free-Fasudil *in vitro*.

Moreover, Lip-Fasudil was found to increase the therapeutic window of Fasudil. In our pilot study, we found that 100 mg/kg Fasudil administrated i.v. caused the death of tumor-bearing mice (data not shown). In contrast, Fasudil at doses lower than 30 mg/kg showed only minor anti-tumor effects (data not shown). Similar results obtained by other groups using Fasudil to treat lung cancer in mice have been reported [16]. These results demonstrate the narrow therapeutic window of Fasudil, which might hinder its clinical application for cancer treatment. However, since Lip-Fasudil reduced the distribution of Fasudil in other normal tissues and hence reduced systemic toxicity, the administrative doses could be escalated when necessary in the clinics. To conclude, our study indicated that Fasudil is effective in inhibiting HCC growth and that Lip-Fasudil can enhance its efficacy and reduce its toxicity. Therefore, liposomal Fasudil holds great potential for use for the treatment of liver cancer.

## Supporting information

S1 Minimal Data Relevant data described in manuscript. (ZIP)
