# Supplementary figures and images for: Enhanced anti-tumor effect of liposomal Fasudil on hepatocellular carcinoma in vitro and in vivo
Source: PLoS One. 2019 Oct 3;14(10):e0223232. doi: 10.1371/journal.pone.0223232 (PMC6776396; doi:10.1371/journal.pone.0223232)

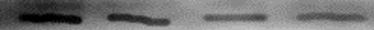

Supplement: S1 Minimal Data — (ZIP) [file pone.0223232.s001.zip › WB/hep3b cycd1.tif]

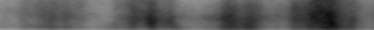

Supplement: S1 Minimal Data — (ZIP) [file pone.0223232.s001.zip › WB/hep3b cycb1.tif]

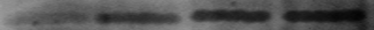

Supplement: S1 Minimal Data — (ZIP) [file pone.0223232.s001.zip › WB/hep3b cyca2.tif]

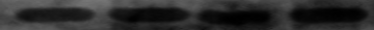

Supplement: S1 Minimal Data — (ZIP) [file pone.0223232.s001.zip › WB/hep3b actin.tif]

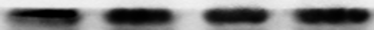

Supplement: S1 Minimal Data — (ZIP) [file pone.0223232.s001.zip › WB/hep3b bax.tif]

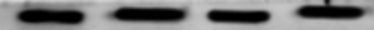

Supplement: S1 Minimal Data — (ZIP) [file pone.0223232.s001.zip › WB/hep3b bcl2.tif]

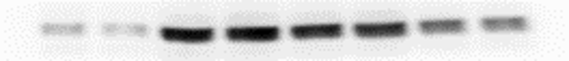

Supplement: S1 Minimal Data — (ZIP) [file pone.0223232.s001.zip › WB/mouse terk.tif]

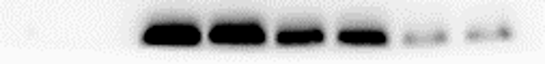

Supplement: S1 Minimal Data — (ZIP) [file pone.0223232.s001.zip › WB/mouse pakt.tif]

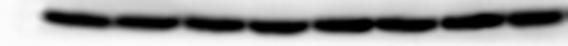

Supplement: S1 Minimal Data — (ZIP) [file pone.0223232.s001.zip › WB/mouse gapdh.tif]

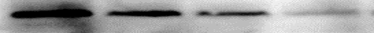

Supplement: S1 Minimal Data — (ZIP) [file pone.0223232.s001.zip › WB/huh7 rock2.tif]

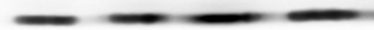

Supplement: S1 Minimal Data — (ZIP) [file pone.0223232.s001.zip › WB/huh7 cyce1.tif]

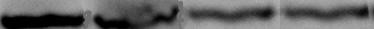

Supplement: S1 Minimal Data — (ZIP) [file pone.0223232.s001.zip › WB/huh7 cycd1.tif]

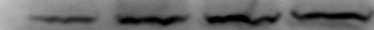

Supplement: S1 Minimal Data — (ZIP) [file pone.0223232.s001.zip › WB/huh7 cycb1.tif]

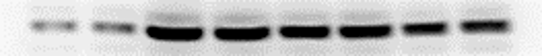

Supplement: S1 Minimal Data — (ZIP) [file pone.0223232.s001.zip › WB/mouse takt.tif]

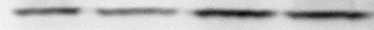

Supplement: S1 Minimal Data — (ZIP) [file pone.0223232.s001.zip › WB/huh7 cyca2.tif]

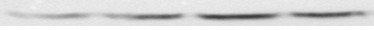

Supplement: S1 Minimal Data — (ZIP) [file pone.0223232.s001.zip › WB/huh7 bcl2.tif]

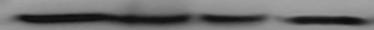

Supplement: S1 Minimal Data — (ZIP) [file pone.0223232.s001.zip › WB/huh7 bax.tif]

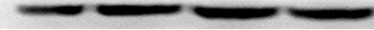

Supplement: S1 Minimal Data — (ZIP) [file pone.0223232.s001.zip › WB/huh7 actin.tif]

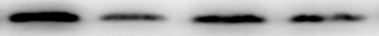

Supplement: S1 Minimal Data — (ZIP) [file pone.0223232.s001.zip › WB/hepg2 rock2.tif]

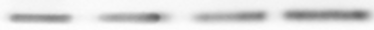

Supplement: S1 Minimal Data — (ZIP) [file pone.0223232.s001.zip › WB/hepg2 cyce1.tif]

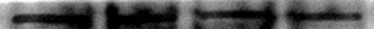

Supplement: S1 Minimal Data — (ZIP) [file pone.0223232.s001.zip › WB/hepg2 cycd1.tif]

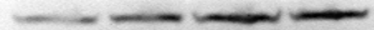

Supplement: S1 Minimal Data — (ZIP) [file pone.0223232.s001.zip › WB/hepg2 cycb1.tif]

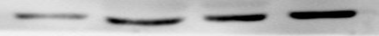

Supplement: S1 Minimal Data — (ZIP) [file pone.0223232.s001.zip › WB/hepg2 cyca2.tif]

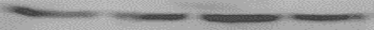

Supplement: S1 Minimal Data — (ZIP) [file pone.0223232.s001.zip › WB/hepg2 bcl2.tif]

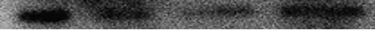

Supplement: S1 Minimal Data — (ZIP) [file pone.0223232.s001.zip › WB/hepg2 bax.tif]

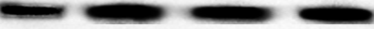

Supplement: S1 Minimal Data — (ZIP) [file pone.0223232.s001.zip › WB/hepg2 actin.tif]

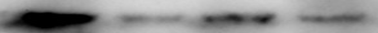

Supplement: S1 Minimal Data — (ZIP) [file pone.0223232.s001.zip › WB/hep3b rock2.tif]

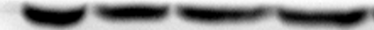

Supplement: S1 Minimal Data — (ZIP) [file pone.0223232.s001.zip › WB/hep3b cyce1.tif]

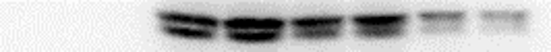

Supplement: S1 Minimal Data — (ZIP) [file pone.0223232.s001.zip › WB/mouse perk.tif]

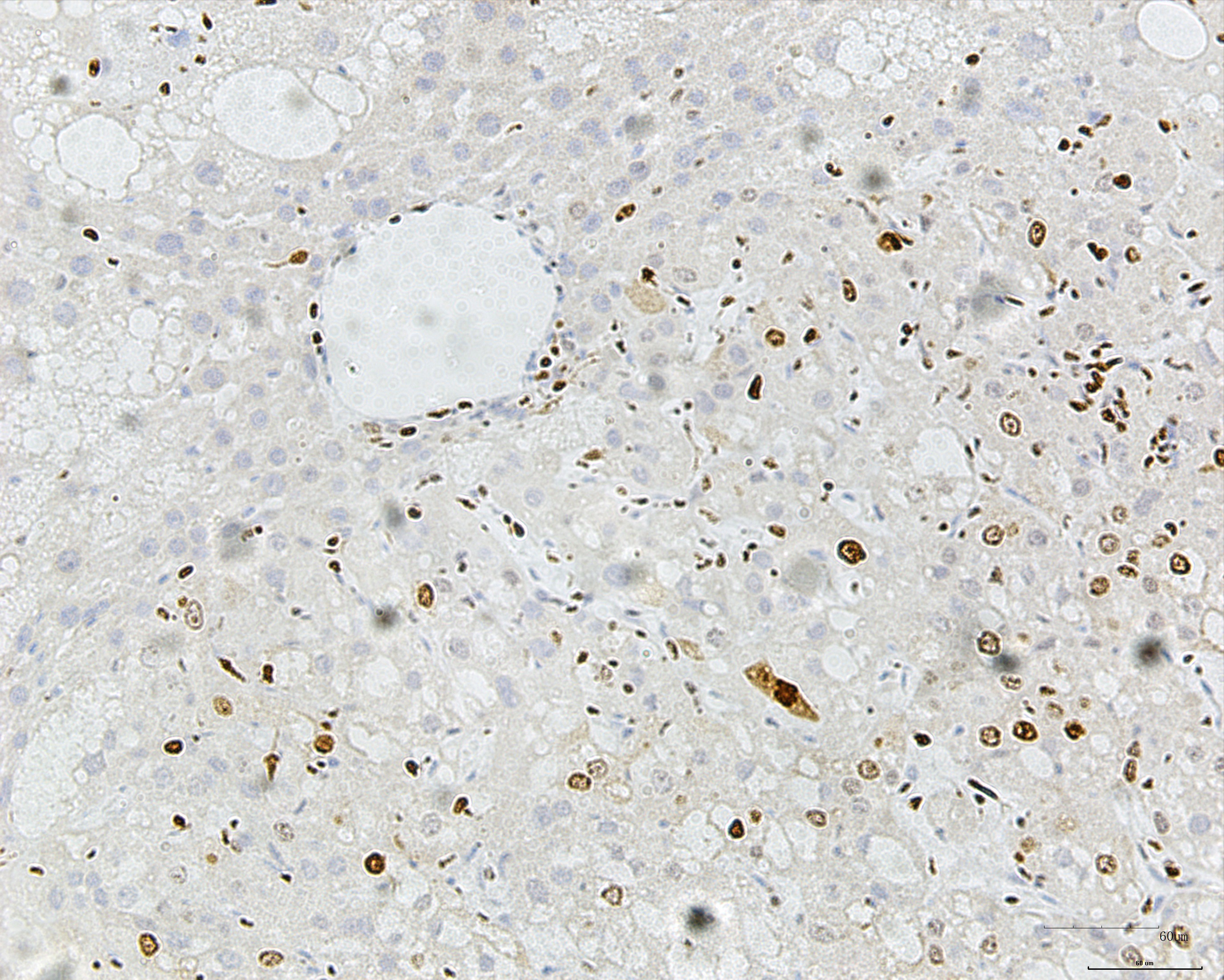

Supplement: S1 Minimal Data — (ZIP) [file pone.0223232.s001.zip › IHC/free fasudil ki67.jpg]

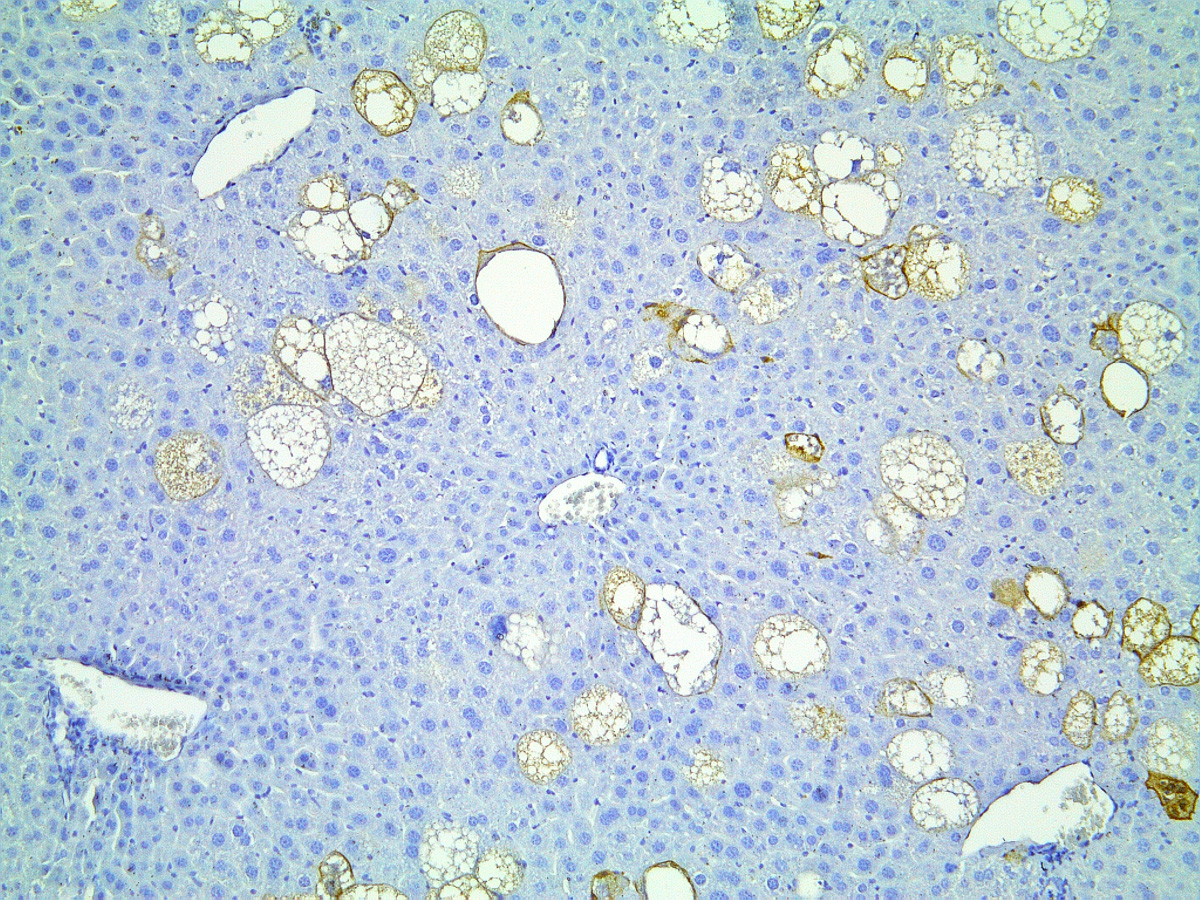

Supplement: S1 Minimal Data — (ZIP) [file pone.0223232.s001.zip › IHC/lip-fasudil HA.jpg]

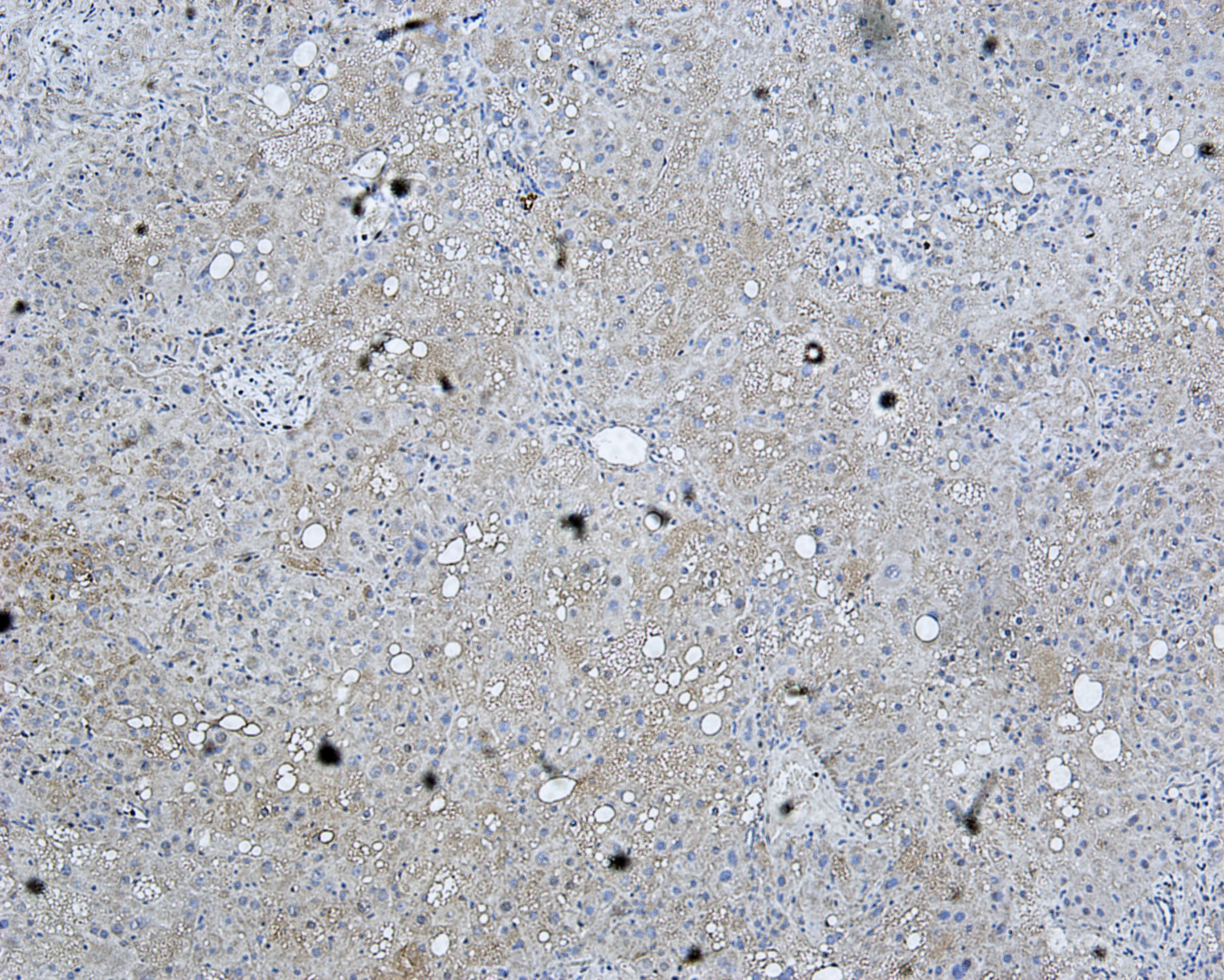

Supplement: S1 Minimal Data — (ZIP) [file pone.0223232.s001.zip › IHC/free fasudil P-AKT.jpg]

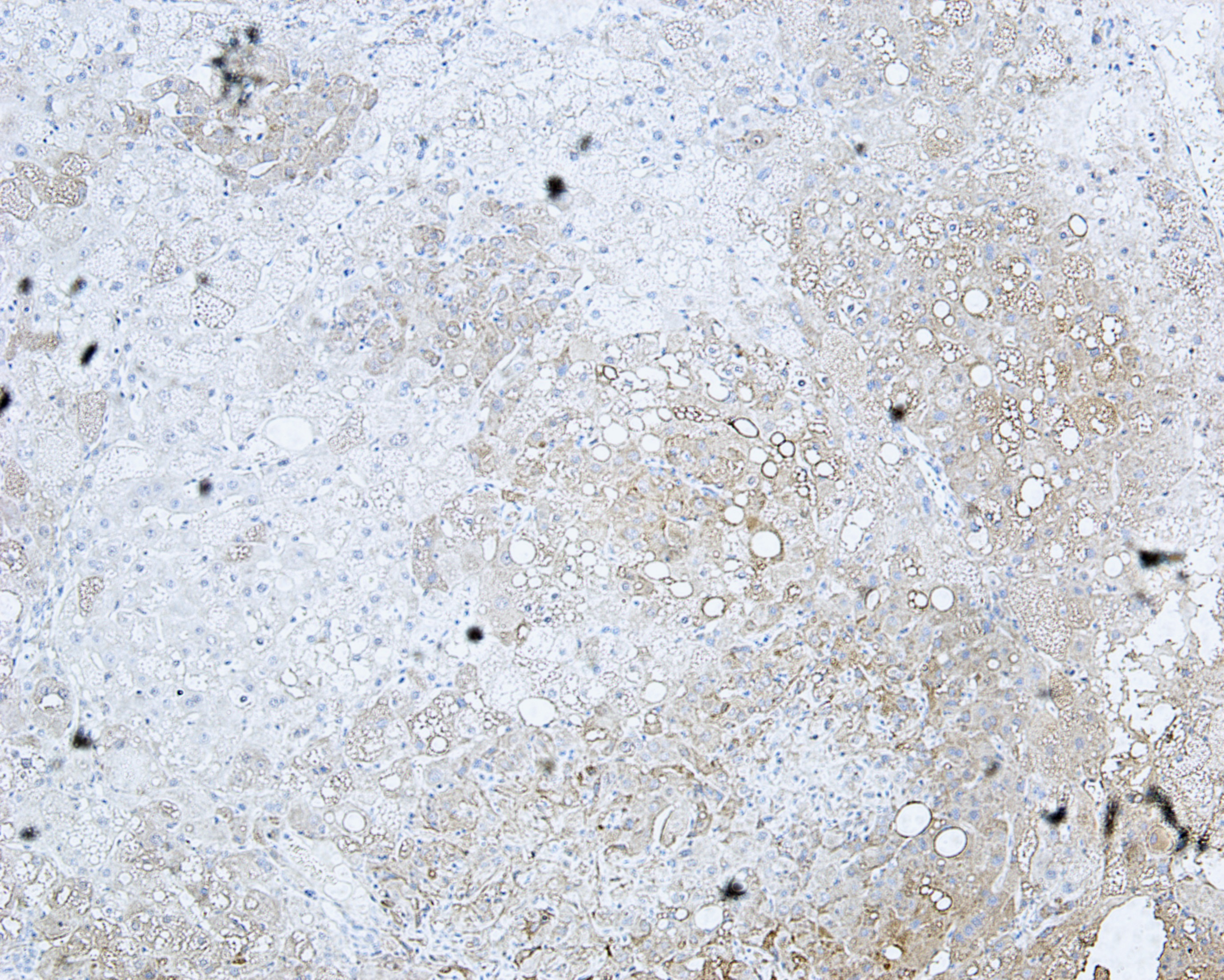

Supplement: S1 Minimal Data — (ZIP) [file pone.0223232.s001.zip › IHC/free fasudil N-RAS.jpg]

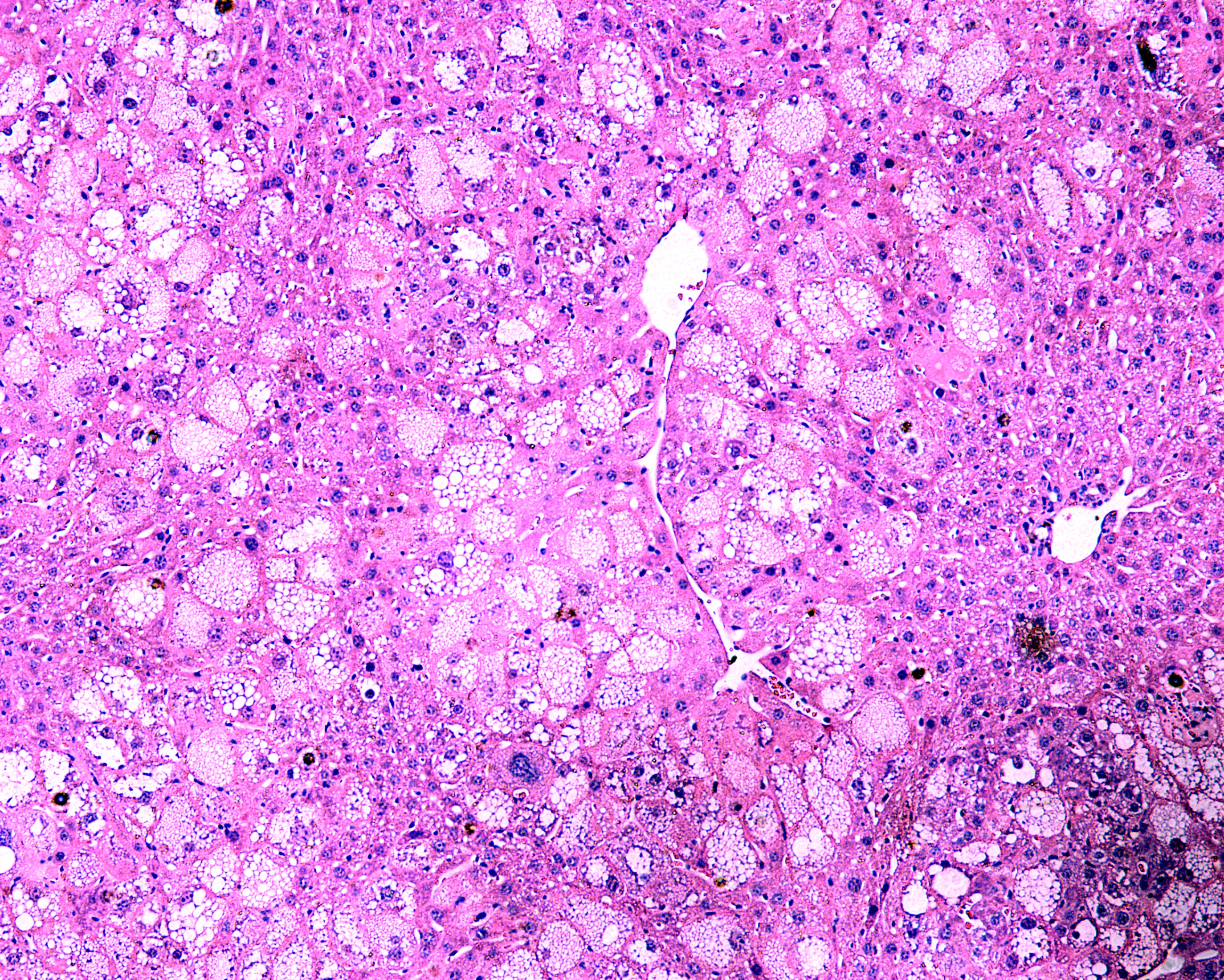

Supplement: S1 Minimal Data — (ZIP) [file pone.0223232.s001.zip › IHC/free fasudil HE.jpg]

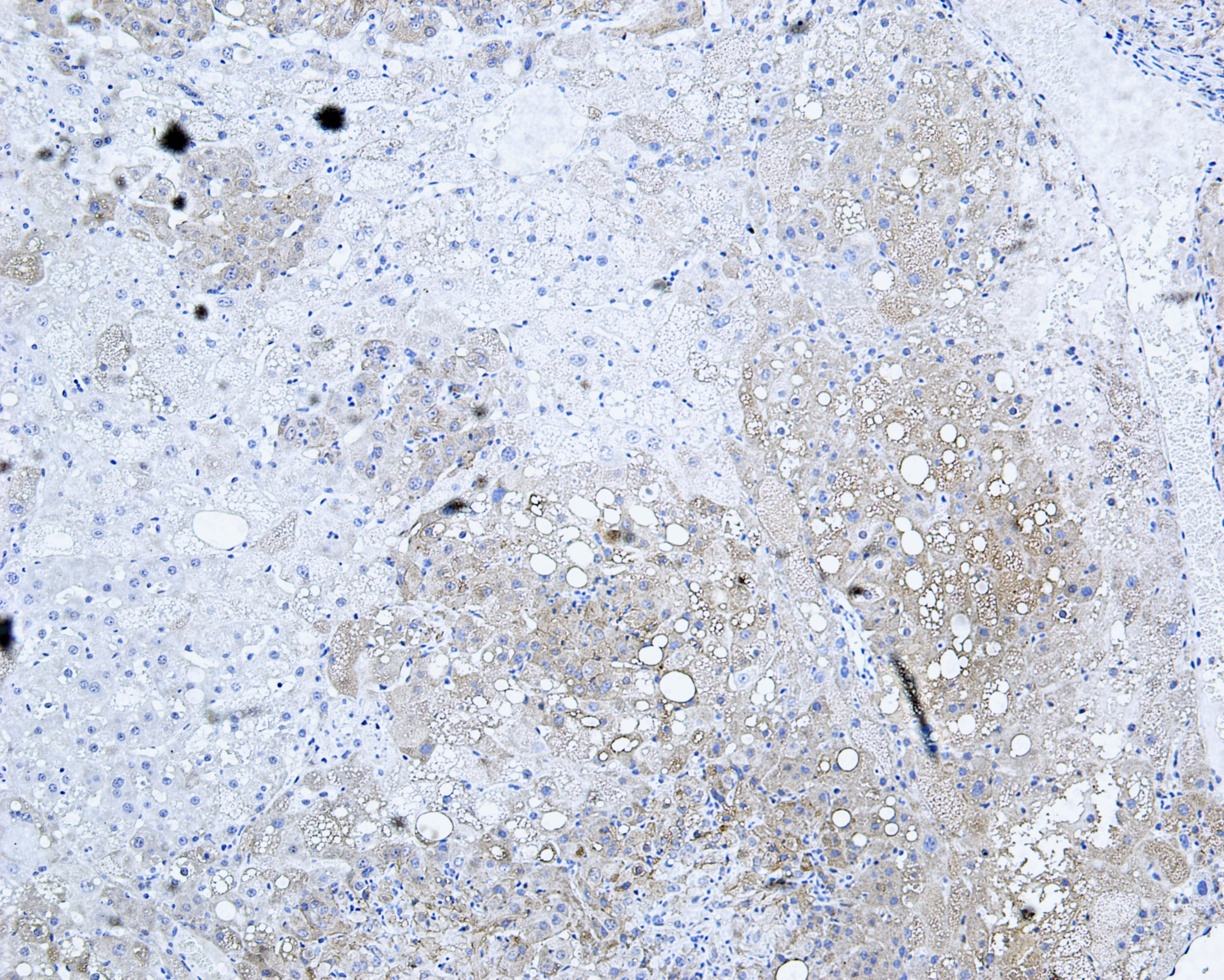

Supplement: S1 Minimal Data — (ZIP) [file pone.0223232.s001.zip › IHC/free fasudil HA.jpg]

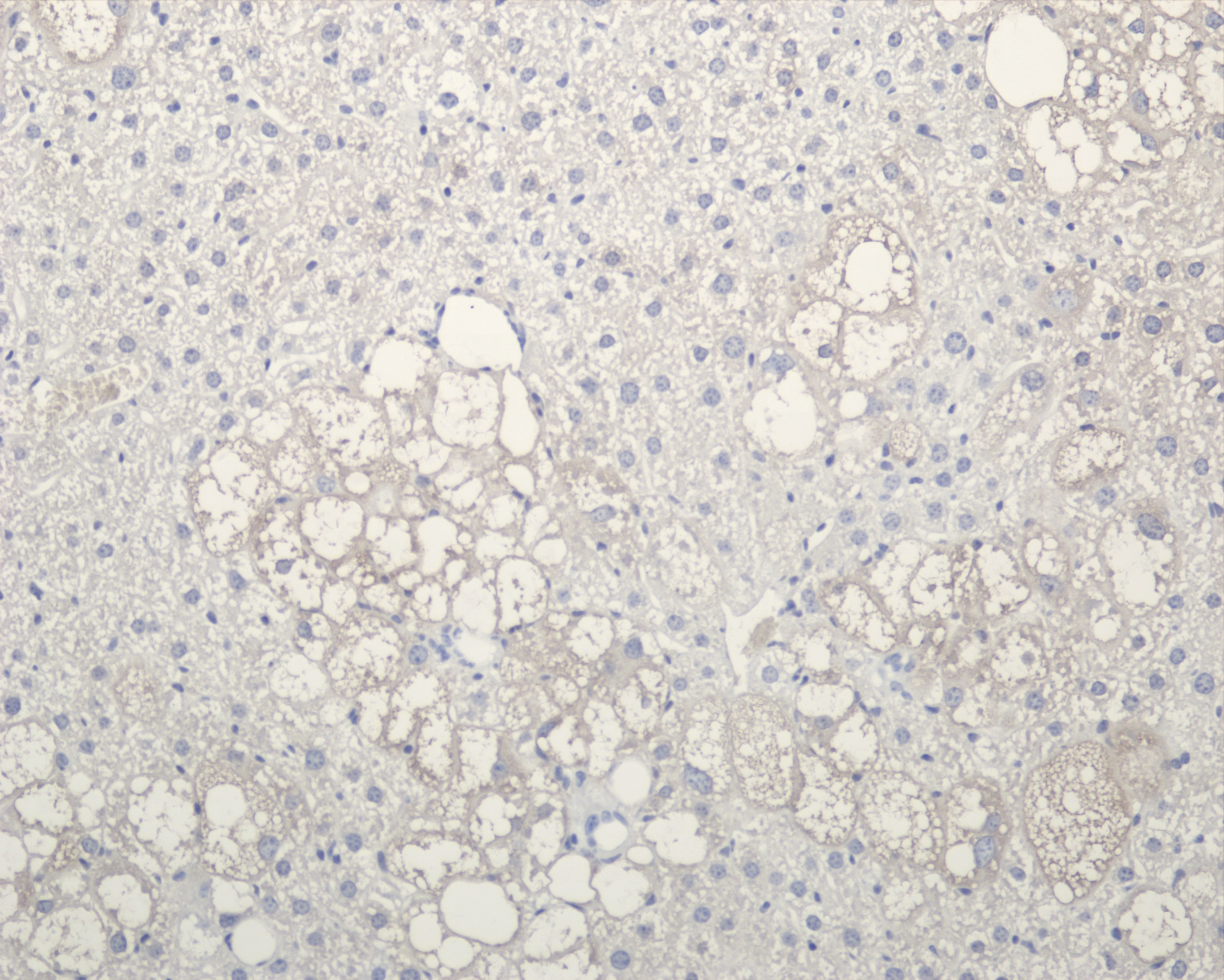

Supplement: S1 Minimal Data — (ZIP) [file pone.0223232.s001.zip › IHC/lip-fasudil N-RAS.jpg]

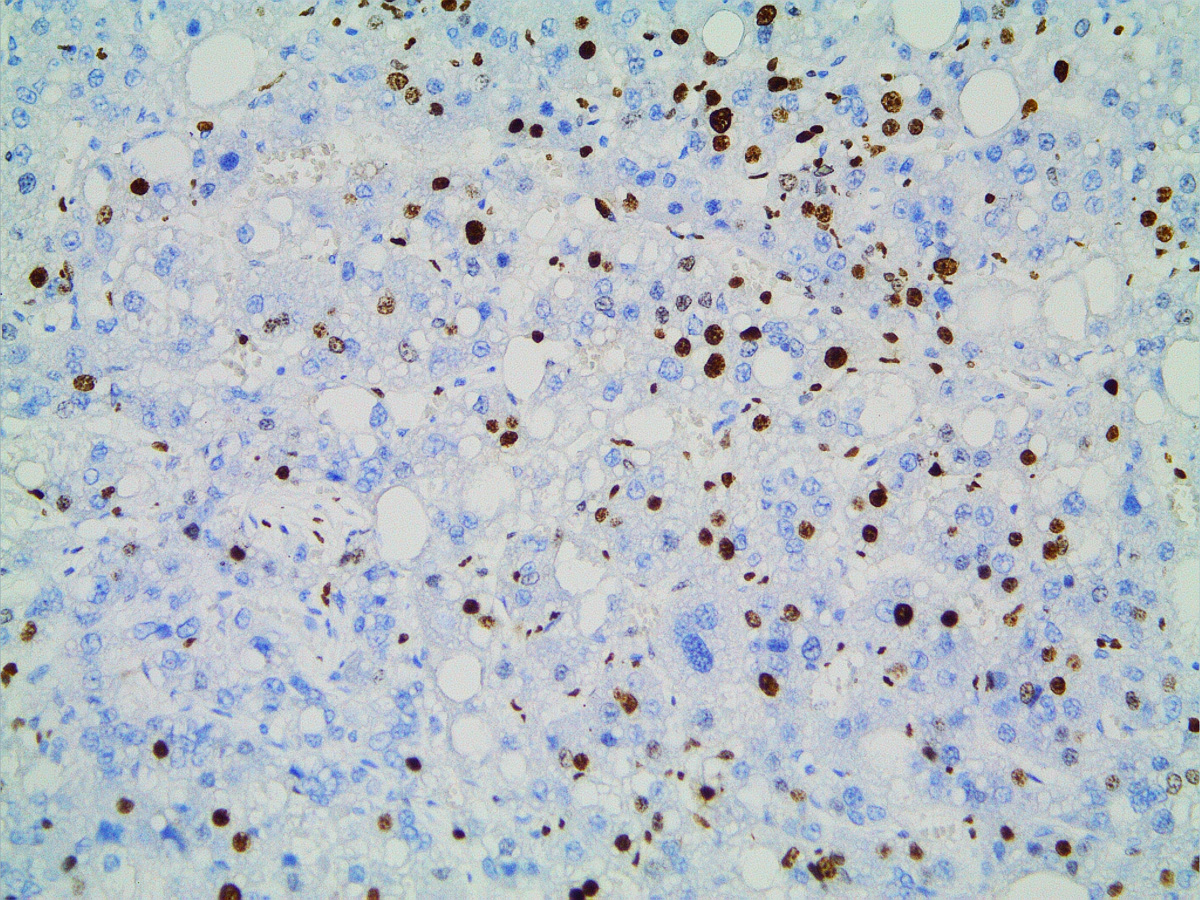

Supplement: S1 Minimal Data — (ZIP) [file pone.0223232.s001.zip › IHC/saline KI67.jpg]

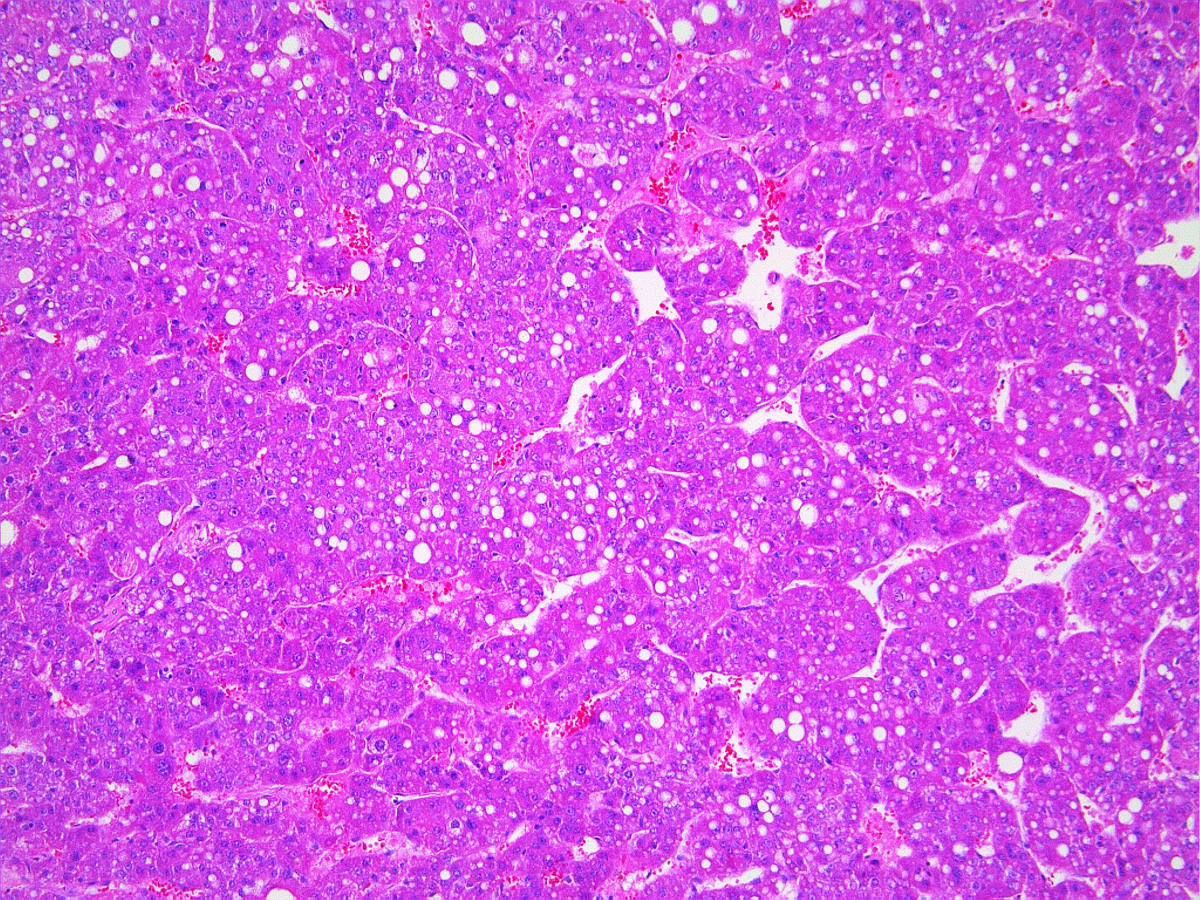

Supplement: S1 Minimal Data — (ZIP) [file pone.0223232.s001.zip › IHC/saline HE.jpg]

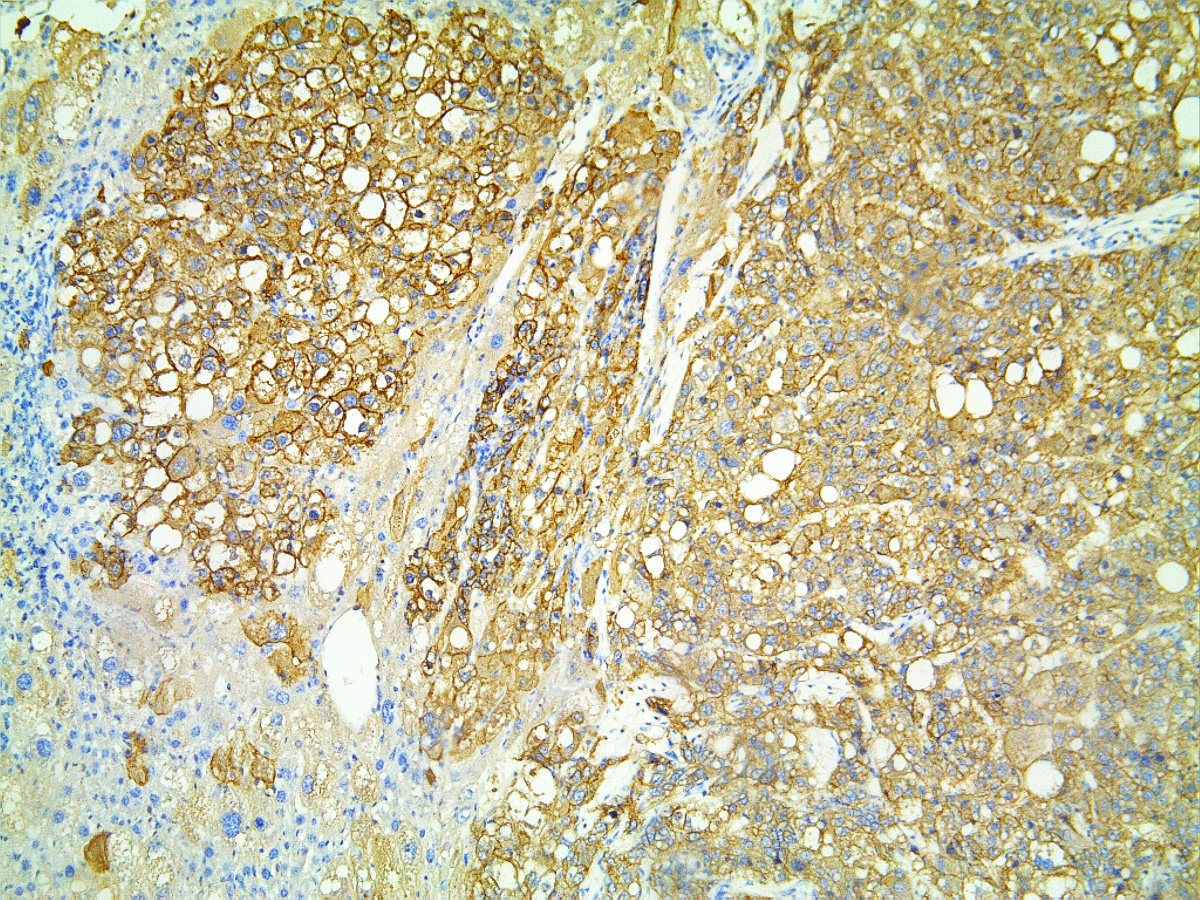

Supplement: S1 Minimal Data — (ZIP) [file pone.0223232.s001.zip › IHC/saline HA.jpg]

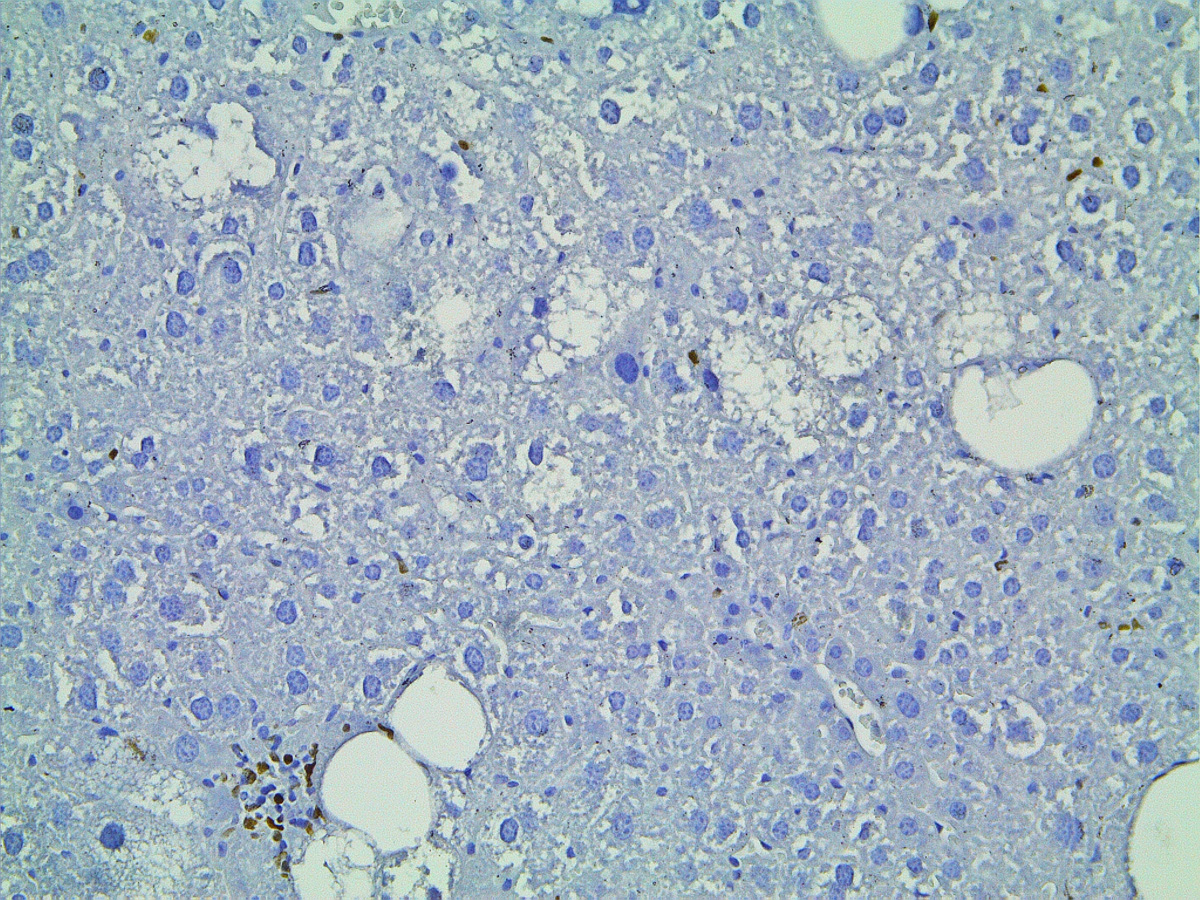

Supplement: S1 Minimal Data — (ZIP) [file pone.0223232.s001.zip › IHC/lip-fasudil ki67.jpg]

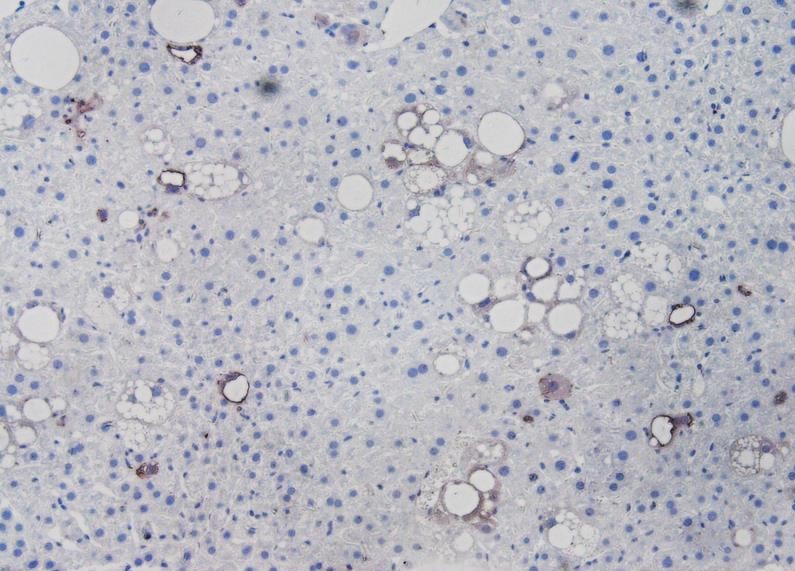

Supplement: S1 Minimal Data — (ZIP) [file pone.0223232.s001.zip › IHC/lip-fasudil P-AKT.jpg]

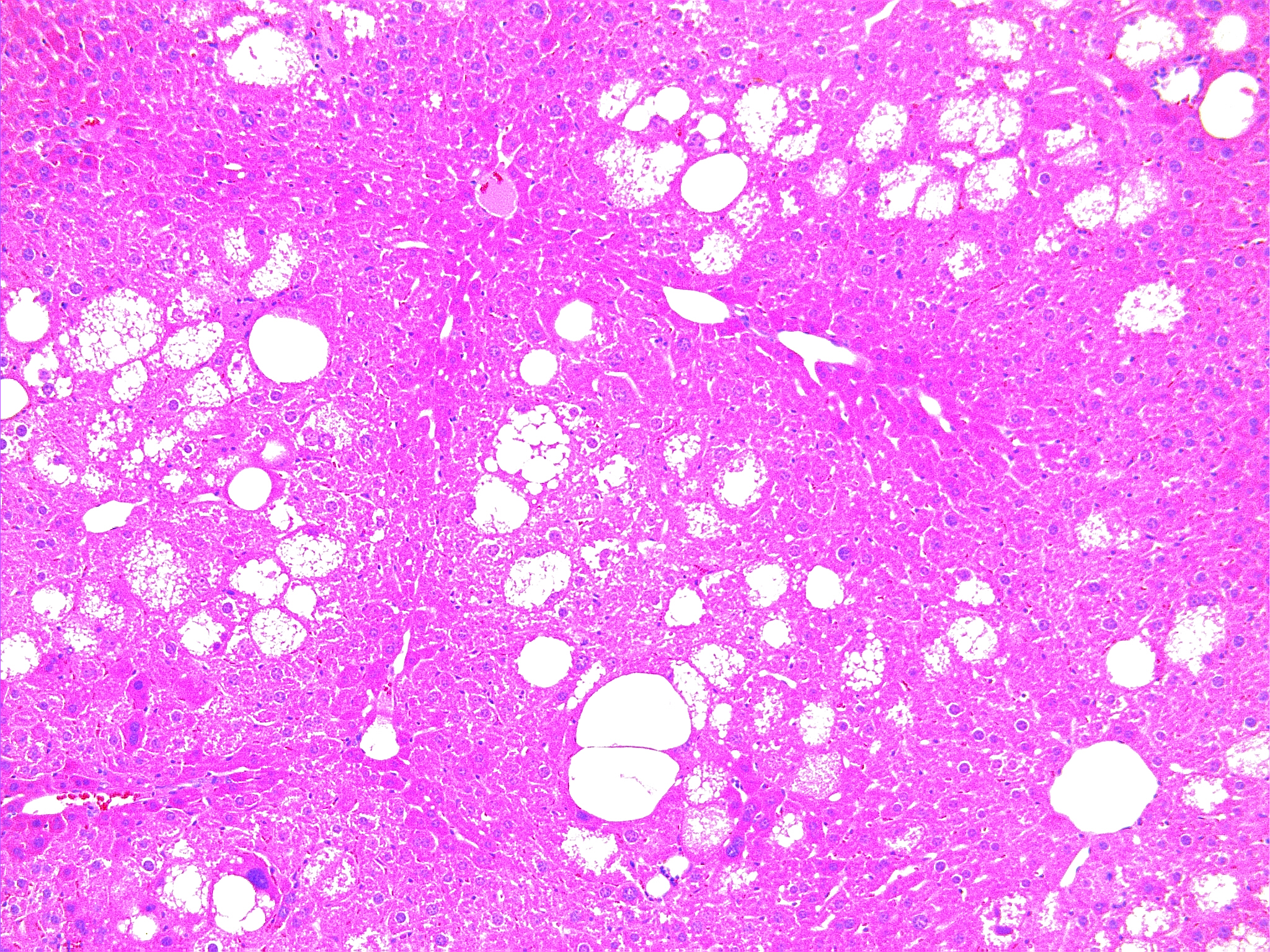

Supplement: S1 Minimal Data — (ZIP) [file pone.0223232.s001.zip › IHC/lip-fasudil HE.jpg]

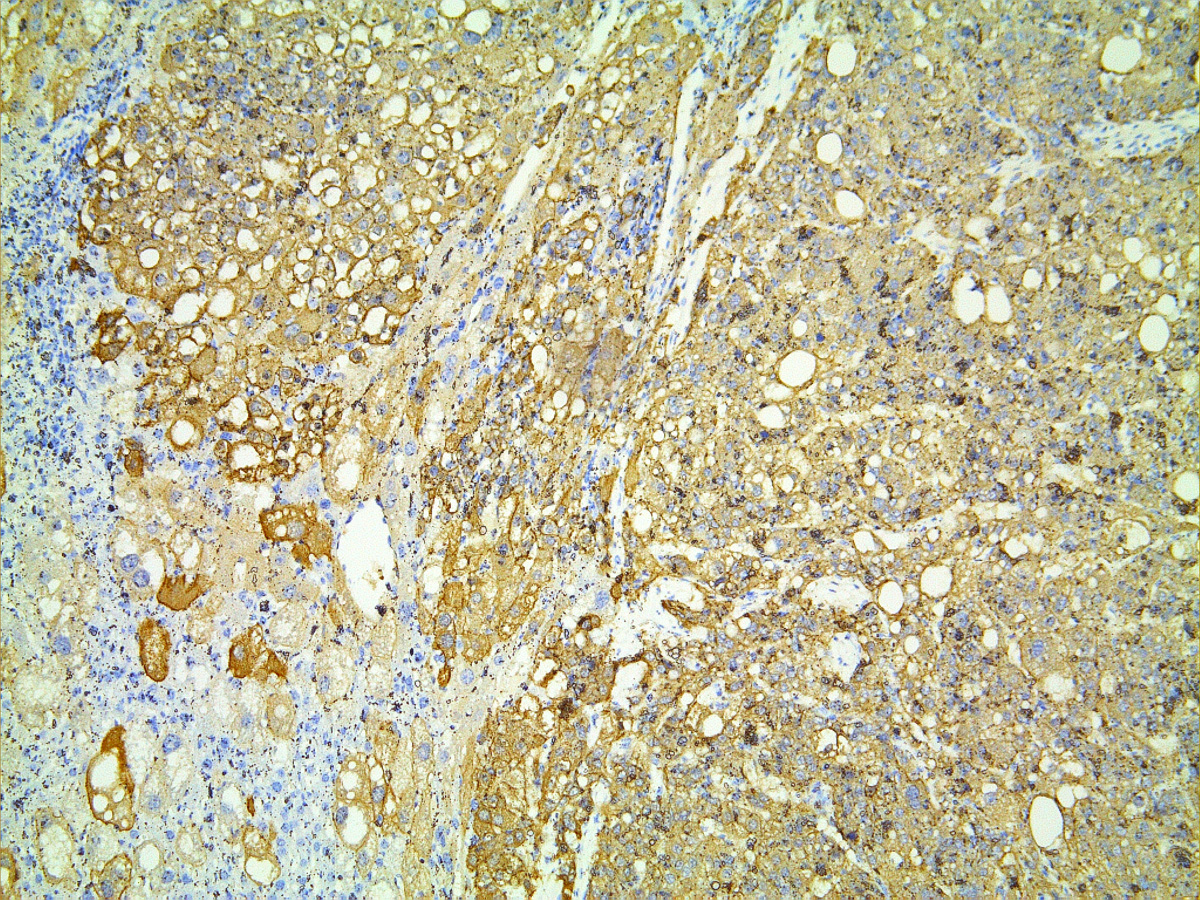

Supplement: S1 Minimal Data — (ZIP) [file pone.0223232.s001.zip › IHC/saline P-AKT.jpg]

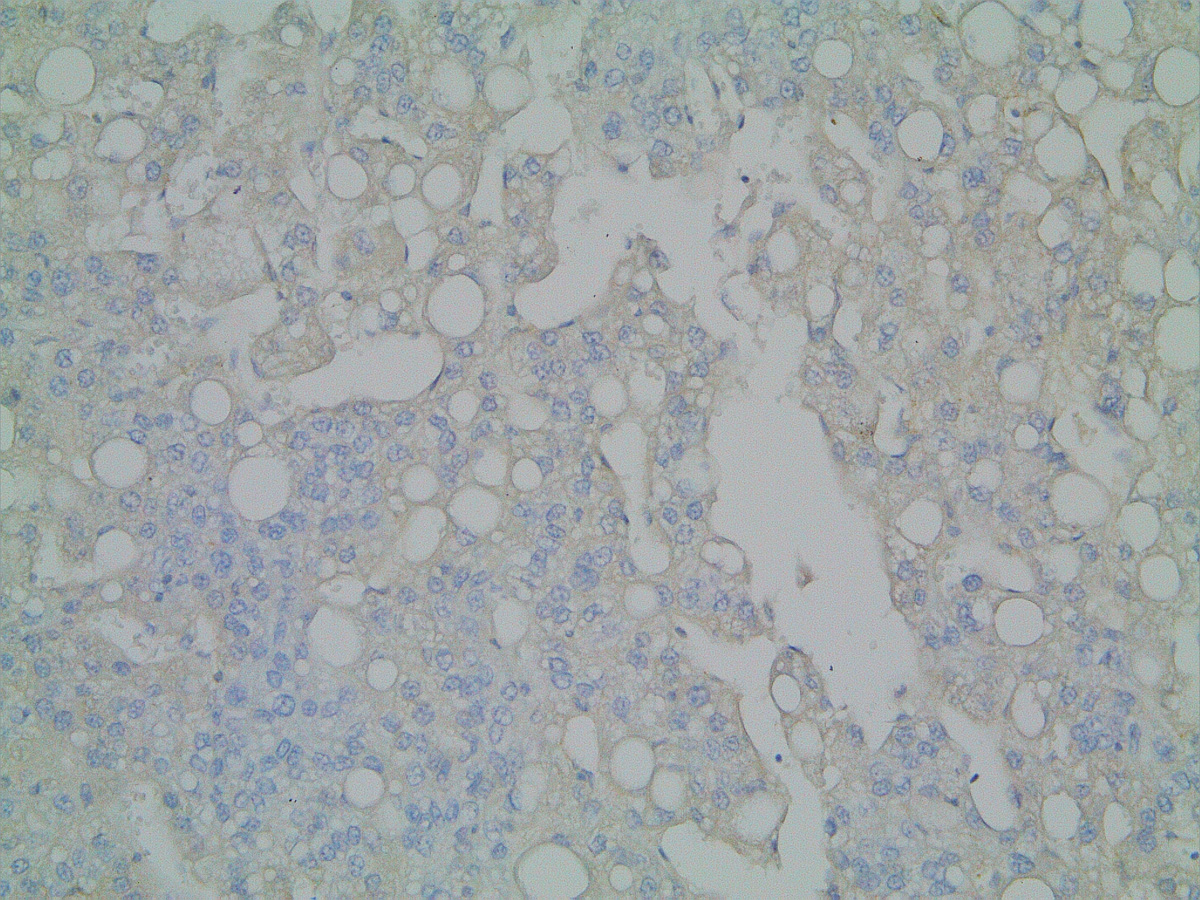

Supplement: S1 Minimal Data — (ZIP) [file pone.0223232.s001.zip › IHC/saline N-RAS.jpg]
